# ZBP1 orchestrates dynamic transitions between cell death pathways in response to arsenic and hyperosmotic stress

**DOI:** 10.1016/j.jbc.2025.110856

**Published:** 2025-10-22

**Authors:** Yinghao Fu, Yifan Yang, Qingqing Li, Xin Xia, Menghan Peng, Shanshan Meng, Rong Shen, Yongyi Zhu, Huipeng Jiao, Chun Kim, Juan Lin

**Affiliations:** 1 State Key Laboratory of Cellular Stress Biology, Cancer Research Center, School of Medicine, Faculty of Medicine and Life Sciences, Xiamen University, Xiamen, China; 2 School of Medicine, Xiamen University, Xiamen, China; 3 Shenzhen Research Institute of Xiamen University, Shenzhen, Guangdong, China; 4 Zhejiang Key Laboratory of Molecular Cancer Biology, Life Sciences Institute, Zhejiang University, Hangzhou, China; 5 Department of Neonatal Surgery, Children's Hospital, Zhejiang University School of Medicine, National Clinical Research Center for Child Health, Hangzhou, China; 6 Department of Medicinal and Life Sciences, Hanyang University [ERICA Campus], Ansan, Republic of Korea; 7 State Key Laboratory of Vaccines for Infectious Diseases, State Key Laboratory of Molecular Vaccinology and Molecular Diagnostics, Center for Molecular Imaging and Translational Medicine, School of Public Health, Xiamen University, Xiamen, China

**Keywords:** apoptosis, necroptosis, pyroptosis, stress granules, ZBP1

## Abstract

Z-DNA binding protein 1 (ZBP1), a sensor of Z-form nucleic acids, plays a crucial role in cell death and inflammation. While its functions in infection and development are well-established, its involvement in environmental stress responses remains largely unexplored. In this study, we uncovered novel mechanisms by which ZBP1 mediates cell death under arsenic and hyperosmotic stress. We demonstrate that ZBP1 initiates necroptosis during the early stages of these stresses. As the stress persists, the cell death mode evolves, shifting towards apoptosis and pyroptosis in later stages. This transition is particularly pronounced when the necroptotic pathway is compromised. Interestingly, despite previous implications of stress granules (SGs) in arsenic-dependent cell death, we found that neither SGs formation inhibitors nor the ablation of SGs components significantly impacts cell death under arsenic or hyperosmotic stress. This suggests that environmental stress-induced necroptosis operates largely independent of SGs formation. Employing genome-wide CRISPR/Cas9 library screening, we identified that ZBP1-dependent cell death in response to arsenic is primarily driven by reactive oxygen species and the KEAP1-NRF2 signaling pathway. Notably, ZBP1 KO mice demonstrated resistance to arsenic-induced cell death and tissue injury, further substantiating our findings. Our research provides important new insights into ZBP1's role in environmental stress-induced cell death, expanding our understanding beyond its established functions in infection and development. These findings offer potential implications for comprehending stress-related tissue injury mechanisms.

Programmed cell death (PCD) plays important roles in physiological and pathological processes (1). Various forms of PCD are activated and regulated by distinct cell signaling cascades in different physiological contexts. Apoptosis, which is important during early development and tissue homeostasis, is a non-inflammatory form of PCD regulated by a cascade of caspases (2). In contrast, necroptosis and pyroptosis are considered as inflammatory cell death. Necroptosis is caspase-independent PCD driven by receptor-interacting serine/threonine protein kinase 3 (RIPK3) (3, 4, 5), which activates pore-forming mixed lineage kinase domain-like protein (MLKL) (6) downstream of multiple signaling pathways. Pyroptosis occurs when activated caspases cleave gasdermins leading to plasma membrane pore formation (7, 8, 9). Although each death pathway is controlled by distinct key regulatory molecules, studies have demonstrated extensive crosstalk between these mechanisms of cell death.

Z-DNA binding protein 1 (ZBP1), also known as DNA-dependent activator of IFN-regulatory factors (DAI), is an interferon-inducible protein containing two tandem Zα domains (10, 11). The Zα domain binds to left-handed Z-form structures of nucleic acids, including Z-DNA and its RNA equivalent, Z-RNA. In addition to the Zα domains, ZBP1 possesses receptor interacting protein homotypic interaction motif (RHIM)s that facilitates the recruitment of other RHIM-containing proteins, such as RIPK1 and RIPK3 (12, 13, 14, 15, 16). ZBP1-mediated necroptosis has been observed in response to certain viral infections or when the *ripk1* gene is mutated or deleted in mice, leading to inflammation (15, 16, 17). Under specific conditions, ZBP1 also appears to induce pyroptosis (18, 19, 20). However, it remains to be determined how ZBP1 is activated under various physiological conditions to trigger different modes of PCD.

When cells are exposed to environmental stresses, such as arsenic or osmotic stress, they halt translation, leading to the formation of cytosolic aggregates called stress granules (SGs) (21, 22). These granules consist of ribosomal subunits, translation initiation factors, mRNAs and RNA-binding proteins (23). The types of RNA-binding proteins recruited to SGs vary depending on the stimuli. For example, G3BP SGs assembly factor 1 (G3BP1) and G3BP SGs assembly factor 2 (G3BP2) are present in SGs induced by oxidative and arsenic stresses but not in those induced by osmotic stress (24). Additionally, ZBP1 is known to co-localize within SGs in response to arsenic and heat shock stresses (25). While the function of ZBP1 during infection and inflammation has been well reported, its physiological role during environmental stress remains largely elusive. In this study, we explore the physiological role of ZBP1 under environmental stress conditions, specifically arsenic stress and hyperosmotic stress. We reveal that arsenic and osmotic stress can promote multiple forms of cell death through ZBP1 activation. Consistently, ZBP1 deficiency protected mice from arsenate-induced tissue damage.

## Results

### Arsenic and osmotic stress induce ZBP1-dependent cell death

**Figure 1:**
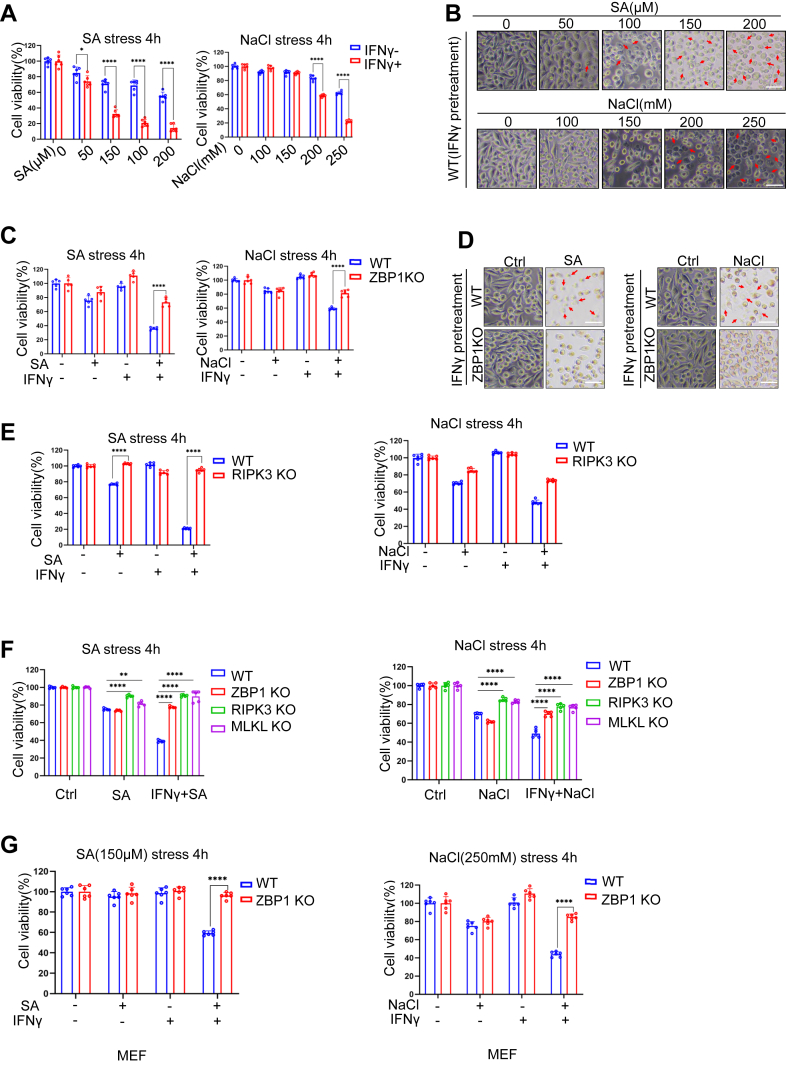
**Arsenic and osmotic stress induce ZBP1-dependent cell death.***A,* WT L929 were treated with different concentrations of sodium arsenite (0, 50, 100, 150, 200 μM) or sodium chloride (0, 100, 150, 200, 250 mM) for 4 h with or without 10 ng ml^−1^ IFNγ pretreatment. Cell viability was determined by neutral red staining (mean values ± SD; Student's *t* test; ∗*p* < 0.05, ∗∗∗∗*p* < 0.0001, n = 2 independent experiments, six replicates per experiment). *B,* microscopic analysis of WT L929 for cell death induced by sodium arsenite or NaCl stress in different concentrations after IFNγ pretreatment. *Arrows* indicate dead cells. Bar, 50 μm. *C,* WT and ZBP1-deficient L929 cells were treated with 100 μM sodium arsenite or 200 mM NaCl for 4 h with or without 10 ng ml^−1^ IFNγ pretreatment. Cell viability was determined by neutral *red* staining (mean values ± SD; Student's *t* test; ∗∗∗∗*p* < 0.0001, n = 3 independent experiments, five or six replicates per experiment). *D,* microscopic analysis of WT or ZBP1-deficient L929 for cell death induced by stress of 100 μM sodium arsenite or 200 mM NaCl for 4 h with or without 10 ng ml^−1^ IFNγ pretreatment. *Arrows* indicate dead cells. Bar, 50 μm. *E,* WT or RIPK3-deficient L929 were treated with 100 μM sodium arsenite or 200 mM NaCl for 4 h with or without 10 ng ml^−1^ IFNγ pretreatment. Cell viability was determined by neutral red staining (mean values ± SD; Student's *t* test; ∗∗∗∗*p* < 0.001, n = 2 independent experiments, six replicates per experiment). *F,* WT, ZBP1-deficient, RIPK3-deficient and mixed lineage kinase domain-like protein (MLKL)-deficient L929 were treated with 100 μM sodium arsenite or 200 mM NaCl for 4 h with or without 10 ng ml^−1^ IFNγ pretreatment. Cell viability was determined by neutral red staining (mean values ± SD; Student's *t* test; ∗∗∗∗*p* < 0.0001, n = 2 independent experiments, five replicates per experiment). *G,* WT and ZBP1-deficient mouse embryonic fibroblast were treated with 100 μM sodium arsenite or 200 mM NaCl for 4 h with or without 10 ng ml^−1^ IFNγ pretreatment. Cell viability was determined by neutral red staining (mean values ± SD; Student's *t* test; ∗∗∗∗*p* < 0.0001, n = 2 independent experiments, six replicates per experiment). MLKL, mixed lineage kinase domain-like protein.

Cells can undergo different forms of cell death when exposed to environmental stresses. To investigate the mode of cell death induced by arsenic and hyperosmotic stress, we used increasing concentrations of sodium arsenate (SA) and hypertonic NaCl solutions to treat L929 cells, which are a mouse fibroblast cell line often used in necroptosis studies. As shown in Figures 1, *A* and *B* and S1*E*, pretreatment of IFNγ on L929 cells markedly enhances SA- or NaCl-induced cell death. However, the effect of IFNγ was largely diminished in ZBP1 KO cells (Figs. 1, *C* and *D*, S1*E*), suggesting that IFNγ-induced ZBP1 plays a significant role in SA- or NaCl-induced cell death.

ZBP1 can interact with RIPK3 to induce cell death (12, 13, 14, 15, 16). To determine whether RIPK3 and its downstream effector MLKL are required for SA- or NaCl-induced cell death, we employed RIPK3- or MLKL-deficient L929 cells. Consistent with our findings in ZBP1-deficient cells, RIPK3 deficiency significantly suppressed SA- or NaCl-induced cell death in the presence of IFNγ (Fig. 1*E*). Notably, the resistance to SA- or NaCl-induced cell death by RIPK3 or MLKL deficiency was more pronounced than that by ZBP1 deficiency (Fig. 1*F*). Furthermore, even in the absence of IFNγ pretreatment, RIPK3- or MLKL-deficient cells exhibited modestly reduced cell death in response to SA or NaCl compared to WT controls (Fig. 1, *E* and *F*). Together, this suggests that RIPK3-MLKL signaling pathway contributes to SA- and NaCl-induced cell death, and its role is amplified in the presence of ZBP1 (Fig. 1, *E* and *F*).

To validate our findings, we performed parallel experiments using mouse embryonic fibroblasts (MEFs). Consistent with our observations in L929 cells, ZBP1-deficient MEFs exhibited increased resistance to SA- and NaCl-induced cell death, particularly when cells were primed with IFNγ (Fig. 1*G*). Collectively, these results collectively establish a critical role for ZBP1 in arsenic and hyperosmotic stress-induced cell death.

### RIPK3 is essential for early necroptotic cell death induced by SA and NaCl

**Figure 2:**
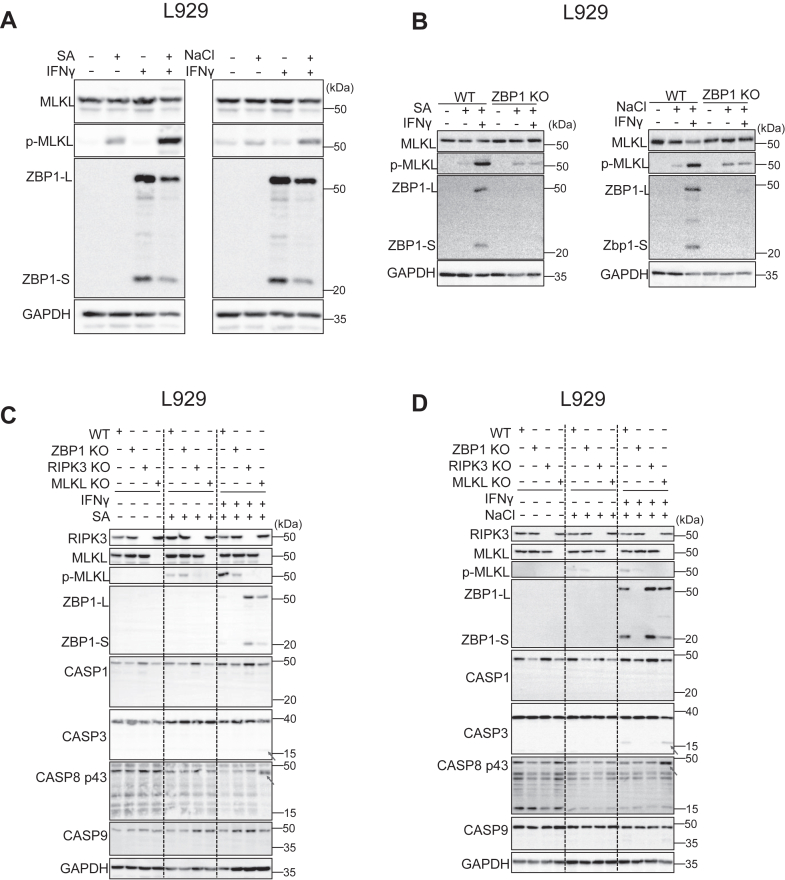
**RIPK3 and MLKL is essential for early necroptotic cell death induced by SA and NaCl in the presence or absence of IFNγ.***A,* immunoblot analysis with indicated antibodies in WT L929 cells stressed with 100 μM sodium arsenite or 200 mM NaCl for 4 h with or without 10 ng ml^−1^ IFNγ pretreatment (n = 2 independent experiments). *B,* immunoblot analysis with indicated antibodies in WT and ZBP1-deficient L929 cells stressed with 100 μM sodium arsenite or 200 mM NaCl for 4 h with or without 10 ng ml^−1^ IFNγ pretreatment (n = 2 independent experiments). *C* and *D,* Immunoblot analysis with indicated antibodies in WT, ZBP1-, RIPK3- and MLKL-deficient L929 cells stressed with 100 μM sodium arsenite or 200 mM NaCl with or without IFNγ pretreatment. *Arrows* indicate cleaved CASP3 (p17) and cleaved CASP8 (p43) (n = 2 independent experiments). MLKL, mixed lineage kinase domain-like protein.

The expression of ZBP1 protein in L929 cells was not detected in untreated cells and also in SA- or NaCl-treated cells. However, it was strongly induced by IFNγ treatment (Fig. 2*A*). We also examined the phosphorylation of MLKL, an ultimate marker of necroptosis, in SA- or NaCl-treated cells. Interestingly, MLKL is phosphorylated upon stimulation with SA or NaCl even in the absence of IFNγ treatment, a condition where ZBP1 protein is not expressed in cells (Fig. 2*A*). This is consistent with the result in Figure 1*F*, where RIPK3- or MLKL-deficient cells showed lower cell death in response to SA or NaCl compared to ZBP1 deficient or WT cells. Nevertheless, the activation of MLKL was prominent when L929 cells were pretreated with IFNγ, and this IFNγ-mediated strong phosphorylation of MLKL markedly disappeared in ZBP1-deficient L929 cells (Figure 2*B*), indicating that ZBP1 contributes to SA- and NaCl-induced cell death in L929 cells in particular when it is induced by IFNγ.

Recent studies reported that in addition to necroptosis, RIPK3 can promote apoptosis or cell death-independent inflammation (26, 27). To investigate the role of RIPK3 in SA- and NaCl-treated cells, we compared signaling events in ZBP1, RIPK3, or MLKL-deficient L929 cells. IFNγ pretreatment followed by 4-h of SA or NaCl stress induced MLKL phosphorylation in WT cells, which was reduced in ZBP1- or RIPK3-deficient cells as expected (Fig. 2, *C* and *D*). Surprisingly, MLKL-deficient cells exhibited modest caspase-8 and -3 activation at this time point (Fig. 2, *C* and *D*). Nevertheless, cell death was significantly inhibited in MLKL-deficient cells at 4 h post-SA or NaCl treatment, indicating that necroptosis is the predominant cell death mechanism at this early time point (Fig. 1, *E* and *F*). Consistent with this observation, immunoprecipitation analysis also confirmed that ZBP1 is associated with RIPK3 and MLKL after IFNγ and SA treatment (Fig. S1*A*).

### ZBP1 mediates a temporal switch in cell death pathways in response to arsenic or hyperosmotic stress

**Figure 3:**
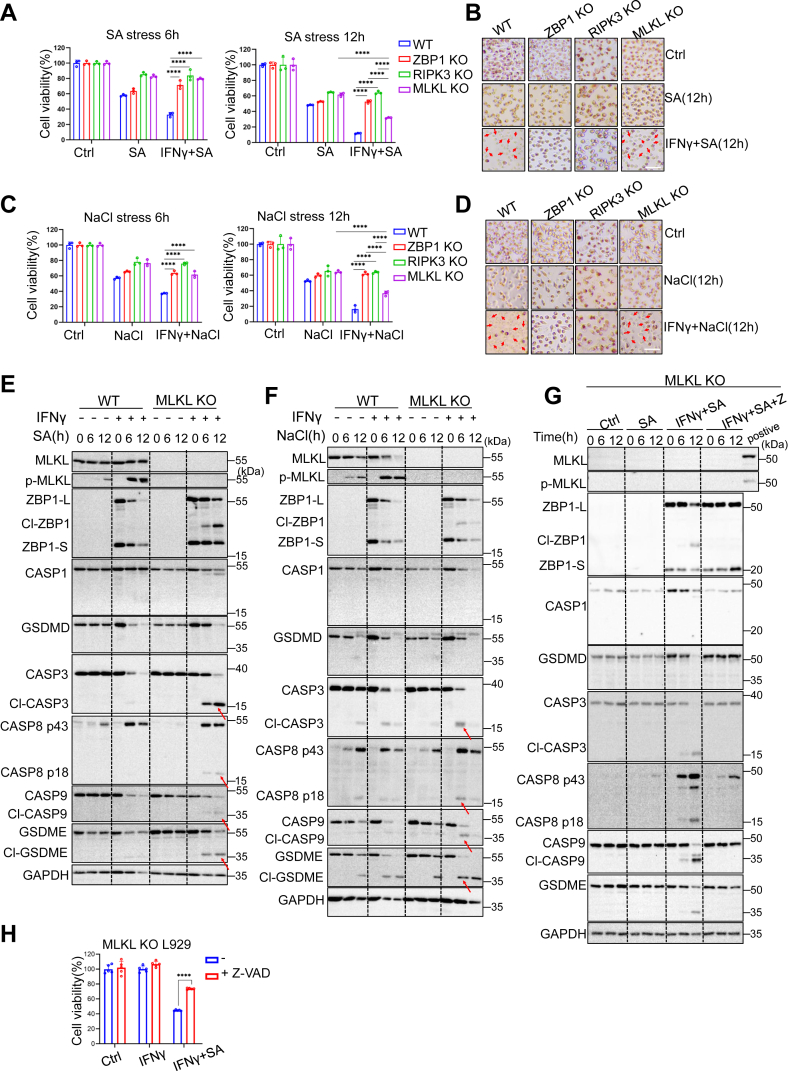
**ZBP1 mediates a temporal switch in cell death pathways in response to arsenic or hyperosmotic Stress.***A* and *B,* WT, ZBP1-, RIPK3- and MLKL-deficient L929 were treated with 100 μM sodium arsenite for 6 and 12 h with or without 10 ng ml^−1^ IFNγ pretreatment. Cell viability was determined by neutral red staining. Representative pics of cell death were shown (mean values ± SD; Student's *t* test; ∗∗∗∗*p* < 0.0001, n = 2 independent experiments, three replicates per experiment). *C* and *D,* WT, ZBP1-, RIPK3- and MLKL-deficient L929 were treated with 200 mM NaCl for 6 and 12 h with or without 10 ng ml^−1^ IFNγ pretreatment. Cell viability was determined by neutral red staining. Representative pics of cell death were shown (mean values ± SD; Student's *t* test; ∗∗∗∗*p* < 0.0001, n = 2 independent experiments, three replicates per experiment). *E* and *F,* immunoblot analysis with indicated antibodies in WT and MLKL-deficient L929 stressed with 100 μM sodium arsenite or 200 mM NaCl with or without IFNγ pretreatment. *Arrows* indicate cleaved CASP3 (p17), cleaved CASP8 (p43), cleaved CASP9 (p37) and cleaved gasdermin E (GSDME) (n = 2 independent experiments). *G,* immunoblot analysis of with indicated antibodies in MLKL-deficient L929 that were treated with 100 μM sodium arsenite with or without IFNγ pretreatment. In addition, Z-VAD-FMK was used to inhibit activation of Caspases (n = 2 independent experiments). *H,* MLKL KO L929 was treated with 100 μM sodium arsenite for 12 h with IFNγ pretreatment. Cell viability was determined by neutral red staining (mean values ± SD; Student's *t* test; ∗∗∗∗*p* < 0.0001, n = 2 independent experiments, five replicates per experiment). In addition, Z-VAD-FMK was used to inhibit activation of Caspases. GSDME, gasdermin E; MLKL, mixed lineage kinase domain-like protein.

Because we observed the initial activations of caspases after 4h of SA or NaCl treatment, we examined if cell death mechanisms evolve over time. To do this, we assessed cell viability at early (6 h) and late (12 h) time points following SA or NaCl treatment. We found that at 6h post stress, MLKL deficiency conferred resistance to cell death similar to RIPK3 deficiency (Fig. 3, *A*–*D*). Unexpectedly, MLKL-deficient cells displayed increased cell death compared to RIPK3- or ZBP1-deficient cells at 12 h, particularly in the presence of IFNγ (Fig. 3, *A*–*D*). To elucidate the underlying signaling mechanisms, we performed immunoblot analysis on WT and MLKL-deficient cells treated with SA or NaCl for 6 and 12 h in the presense or absence of IFNγ. MLKL phosphorylation and caspase-8 cleavage were detected in WT cells, and these signaling processes were enhanced by IFNγ (Fig. 3, *E* and *F*). In contrast, MLKL-deficient cells displayed robust activation of caspases-3, 8, and 9, particularly in the presence of IFNγ. Notably, caspase activation was correlated with ZBP1 and gasdermin E (GSDME) cleavage (Fig. 3, *E* and *F*).

Given that activated caspase-3 cleaves GSDME to induce pyroptosis (28), we treated MLKL-deficient cells with the pan-caspase inhibitor zVAD. Inhibition of caspases prevented not only the cleavage of GSDME and ZBP1 but also cell death induced by SA in the presence of IFNγ in MLKL KO L929 cells (Fig. 3, *G* and *H*). To examine the hierarchy of caspase activation during ZBP1-dependent cell death in our experimental model, we generated cells with targeted KOs of caspase-3, -8, and -9 in an MLKL-deficient background. Our results (Figs. S2, *A*–*D*, S3*A*) reveal several key findings. First, Caspase-8 plays a dominant role. SA and NaCl-induced cell death was more significantly protected in caspase-8 KO cells compared to caspase-3 or caspase-9 KO cells (Fig. S2, *A*–*D*). To further assess mitochondrial pathway involvement, we performed JC-1 staining to monitor membrane potential (Fig. S3*A*). JC-1 fluoresces red in intact mitochondria with high membrane potential, but shifts to green when the potential is lost, indicating mitochondrial damage. As shown in Figure S3, MLKL KO cells treated with either IFNγ+SA or IFNγ+NaCl accumulated green fluorescence, consistent with mitochondrial dysfunction. Notably, MLKL/CASP8 double knockout (DKO) markedly reduced JC-1 monomerization, suggesting that caspase-8 activation is upstream of the intrinsic mitochondrial pathway. Collectively, these findings demonstrate that in ZBP1-expressing cells, arsenic or hyperosmotic stress initially induces necroptosis, followed by a caspase-8-mediated switch to apoptosis and pyroptosis when necroptosis is inhibited.

### RIPK1 and RIPK3 promote ZBP1-mediated apoptosis and pyroptosis in the absence of necroptosis

**Figure 4:**
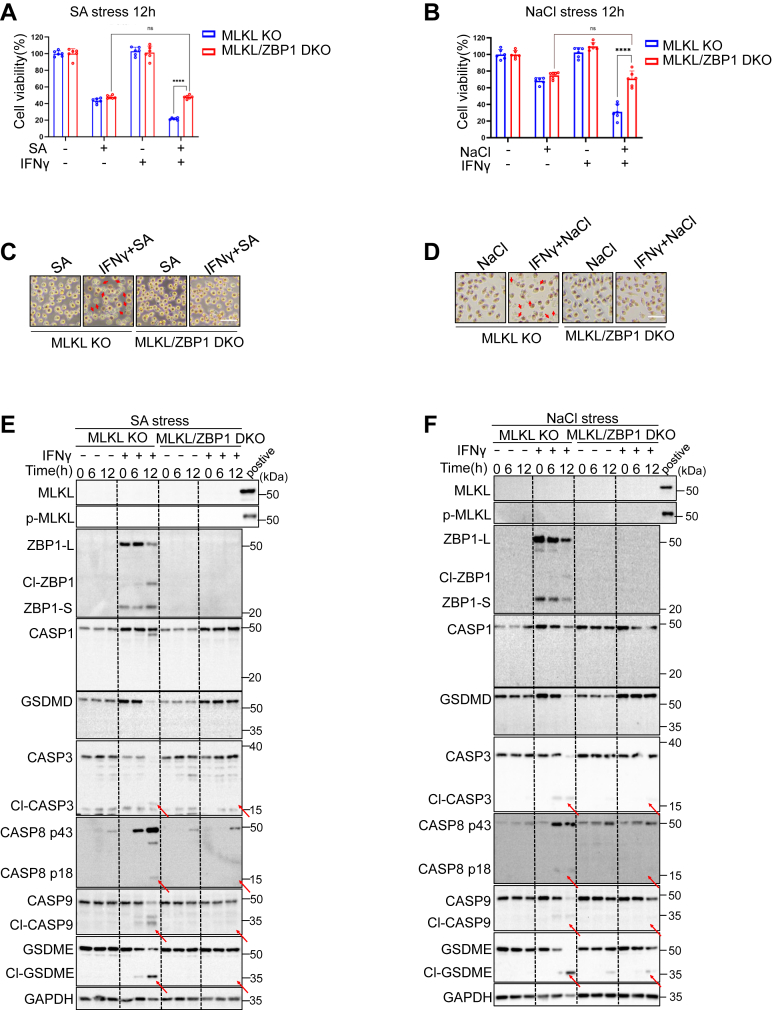
**ZBP1 mediated apoptosis and pyroptosis in the absence of necroptosis.***A*–*D,* MLKL KO and MLKL/ZBP1 DKO L929 were treated with 100 μM sodium arsenite or 200 mM NaCl for 12 h with or without 10 ng ml^−1^ IFNγ pretreatment. Cell viability was determined by neutral red staining (mean values ± SD; Student's *t* test; ∗∗∗∗*p* < 0.0001, n = 2 independent experiments, six replicates per experiment). *E* and *F,* immunoblot analysis with indicated antibodies in MLKL KO and MLKL/ZBP1 DKO L929 that were treated with 100 μM sodium arsenite or 200 mM NaCl with or without IFNγ pretreatment. *Arrows* indicate cleaved CASP3 (p17), cleaved CASP8 (p43), cleaved CASP9 (p37) and cleaved GSDME (n = 2 independent experiments). GSDME, gasdermin E; MLKL, mixed lineage kinase domain-like protein.

To elucidate the interplay between key components of the identified ZBP1-dependent cell death pathways in response to SA or NaCl, we generated MLKL/ZBP1, MLKL/RIPK3, and MLKL/RIPK1 DKO cells (Fig. S1, *B* and *C*). We observed that ZBP1 deficiency significantly rescued MLKL-deficient cells from enhanced cell death induced by the combination of IFNγ plus SA or NaCl, restoring cell death levels to those observed with SA or NaCl alone (Fig. 4, *A*–*D*). In consistent with the data, caspases and GSDME activation induced by IFNγ plus SA, or NaCl in MLKL-deficient cells was markedly reduced in MLKL/ZBP1 DKO cells (Fig. 4, *E* and *F*).

**Figure 5:**
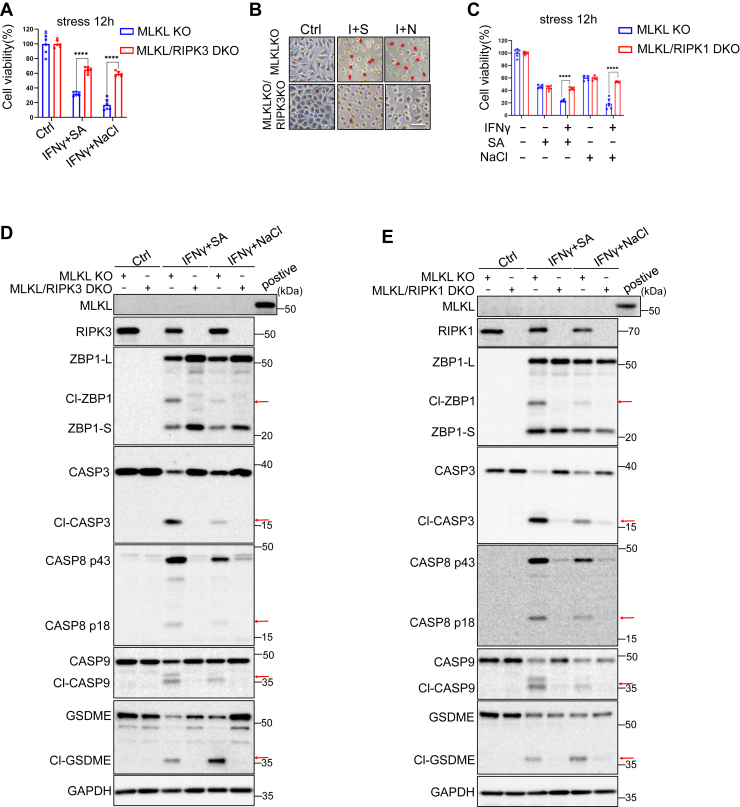
**RIPK1 and RIPK3 promote ZBP1-mediated apoptosis and pyroptosis in the absence of necroptosis.***A* and *B,* MLKL KO and MLKL/RIPK3 DKO L929 were stressed with 100 μM sodium arsenite or 100 μM sodium arsenite for 12 h with 10 ng ml^−1^ IFNγ pretreatment. Cell viability was determined by neutral red staining (mean values ± SD; Student's *t* test; ∗∗∗∗*p* < 0.0001, n = 2 independent experiments, six replicates per experiment). *C,* MLKL KO and MLKL/RIPK1 DKO L929 were treated with 100 μM sodium arsenite or 100 μM sodium arsenite for 12 h with or without 10 ng ml^−1^ IFNγ pretreatment. Cell viability was determined by neutral red staining (mean values ± SD; Student's *t* test; ∗∗∗∗*p* < 0.0001, n = 2 independent experiments, six replicates per experiment). *D,* immunoblot analysis with indicated antibodies in MLKL KO and MLKL/RIPK3 DKO L929 stressed with 100 μM sodium arsenite or 200 mM NaCl for 12 h with or without IFNγ pretreatment. *Arrows* indicate cleaved ZBP1, cleaved CASP3 (p17), cleaved CASP8 (p43), cleaved CASP9 (p37) and cleaved GSDME. *E,* immunoblot analysis with indicated antibodies in MLKL KO and MLKL/RIPK1 DKO L929 that were treated with 100 μM sodium arsenite or 200 mM NaCl for 12 h with or without IFNγ pretreatment. *Arrows* indicate cleaved ZBP1, cleaved CASP3 (p17), cleaved CASP8 (p43), cleaved CASP9 (p37) and cleaved GSDME (n = 2 independent experiments). GSDME, gasdermin E; MLKL, mixed lineage kinase domain-like protein.

Similarly, RIPK3 or RIPK1 deficiency also attenuated the exacerbated cell death in MLKL-deficient cells exposed to IFNγ with the combination of SA, or NaCl (Fig. 5, *A*–*C*). Moreover, the cleavage of ZBP1, caspases-3, 8, 9, and GSDME in MLKL-deficient cells was prevented in MLKL/RIPK3 or MLKL/RIPK1 DKO cells (Fig. 5, *D* and *E*). These findings indicate that SA or NaCl induce ZBP1-dependent cell death signaling, which is mediated by RIPK1 and RIPK3. Inhibition of necroptosis, such as through MLKL ablation, promotes apoptosis and pyroptosis signaling pathways controlled by both RIPK3 and RIPK1 (Fig. S1*D*).

### SGs formation is dispensable for arsenic- and hyperosmotic stress-induced cell death

**Figure 6:**
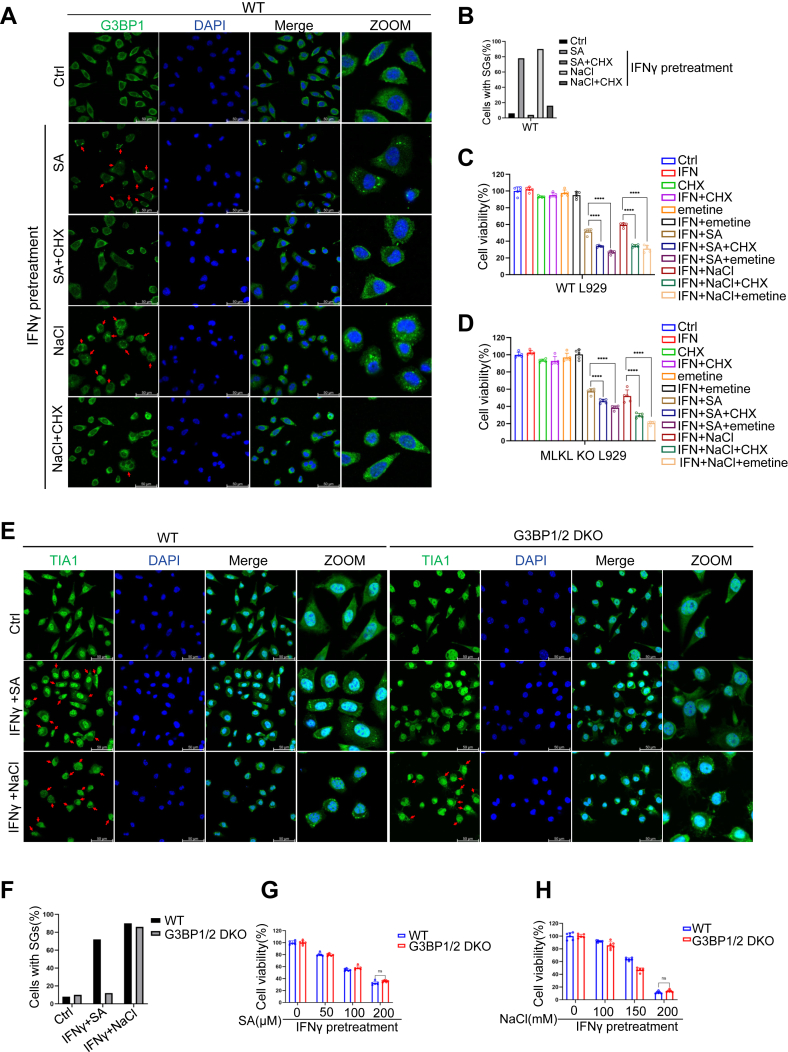
**Stress granulesformation is dispensable for arsenic- and hyperosmotic stress-induced cell death.***A* and *B,* representative images of cells stained with DAPI or immunostained for G3BP1 and graphs depicting quantification of the proportion of G3BP1^+^ cells in IFNγ primed WT L929 cells stressed with 100 μM sodium arsenite or 200 mM NaCl for 2 h with or without 50 μg ml^−1^ cycloheximide pretreatment. *Arrows* indicate cells that form G3BP1 aggregation, which is a characterization of stress granules formation. Bar, 50 μm (n = 2 independent experiments). *C* and *D,* IFNγ primed L929 were stressed with 100 μM sodium arsenite or 200 mM NaCl for 4 h (WT) or 8 h (MLKL KO) with or without 50 μg ml^−1^ cycloheximide pretreatment. Cell viability was determined by neutral red staining (mean values ± SD; Student's *t* test; ∗∗∗*p* < 0.001, ∗∗∗∗*p* < 0.0001, n = 2 independent experiments, six replicates per experiment). *E* and *F,* Representative images of cells stained with DAPI or immunostained for TIA1 and graphs depicting quantification of the proportion of TIA1^+^ cells in IFNγ primed WT or G3BP1/2 DKO L929 cells stressed with 100 μM sodium arsenite or 200 mM NaCl for 2 h. *Arrows* indicate cells that form TIA1 aggregation, which is a characterization of stress granules formation. Bar, 50 μm (n = 2 independent experiments). *G* and *H,* IFNγ primed WT or G3BP1/2 DKO L929 were stressed with sodium arsenite or NaCl of different concentrations for 4 h Cell viability was determined by neutral red staining (mean values ± SD; Student's *t* test; ns *p* > 0.5, n = 2 independent experiments, six replicates per experiment). MLKL, mixed lineage kinase domain-like protein.

Previous studies have reported that SGs can be recognized by ZBP1, leading to necroptosis (29, 30). Given that arsenic and hyperosmotic stress induce SGs formation (24, 25), we investigated the potential role of SGs in our cell death model. To assess the involvement of SGs, we examined the localization of ZBP1 and the SGs component protein, G3BP1. Both proteins co-localized in cytoplasmic SGs formed in response to IFNγ and SA, or IFNγ and NaCl treatment in both WT and MLKL-deficient cells (Fig. S4*A*). Surprisingly, inhibition of SGs formation using cycloheximide (CHX) or emetine abolished SA- or NaCl-induced SGs but failed to prevent cell death in either WT or MLKL-deficient cells (Figs. 6, *A*–*D*, S5, *A*–*E*). Contrary to expectations, we observed that inhibiting SGs formation with CHX or emetine actually led to an increase of cell death, where such an effect was discernible (Fig. 6, *C* and *D*). To further explore the role of SGs in cell death, we utilized G3BP1/2-deficient cells (Fig. S5*F*). These cells failed to form SGs in response to IFNγ and SA, as confirmed by the absence of TIA1 aggregation (Fig. 6, *E* and *F*). However, IFNγ/SA-induced cell death was unaffected by G3BP1/2 deficiency (Fig. 6*G*). IFNγ/NaCl induced SGs formation was not dependent on G3BP1/2 (Fig. 6, *E* and *F*), consistent with previous findings (24). Moreover, hyperosmotic stress-induced cell death was unaffected by G3BP1/2 deficiency (Fig. 6*H*). Collectively, these results demonstrate that SGs formation is not essential for arsenic- or hyperosmotic stress-induced cell death.

### KEAP1-NRF2 axis protects against arsenic-induced cell death independent of SGs formation

**Figure 7:**
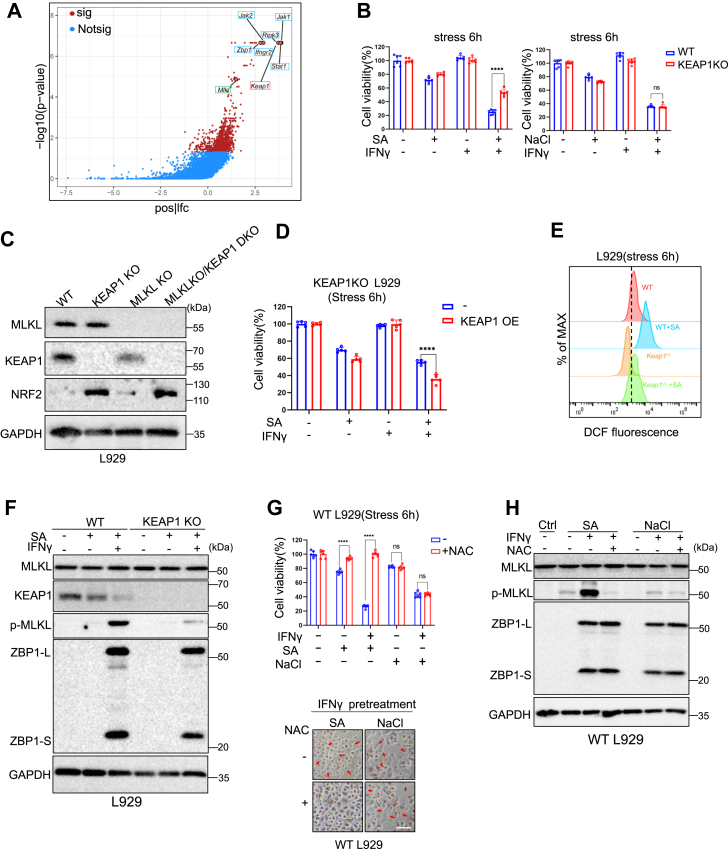
**KEAP1-NRF2 Axis protects against arsenic-induced cell death.***A,* the visualization analysis of CRISPR screening results. The red dots indicate significance gene (−log10 > 1.3), while the blue dots indicate insignificance gene (−log10 < 1.3). Sig: significance, Notsig: not significance, pos|lfc: positive|log2 fold change. *B,* WT or KEAP1 KO L929 was stressed with 100 μM sodium arsenite or 200 mM NaCl for 6 h with or without IFNγ pretreatment. Cell viability was determined by neutral red staining (mean values ± SD; Student's *t* test; ns *p* > 0.1, ∗∗∗∗*p* < 0.0001, n = 2 independent experiments, six replicates per experiment). *C,* immunoblotting analysis with indicated antibodies in WT, KEAP1-deficient, MLKL-deficient and MLKL/KEAP1 DKO L929. *D,* KEAP1-deficient L929 cells reconstituted with or without KEAP1 were stressed with 100 μM sodium arsenite for 6 h with or without IFNγ pretreatment (mean values ± SD; Student's *t* test; ∗∗∗∗*p* < 0.0001 n = 2 independent experiments, five replicates per experiment). *E,* DCFH-DA staining of WT or KEAP1-deficient L929 those were stress with 100 μM sodium arsenite for 6 h to evaluate intracellular ROS levels. *F,* immunoblot analysis with indicated antibodies in WT or KEAP-deficient L929 stressed with 100 μM sodium arsenite for 6 h with or without IFNγ pretreatment (n = 2 independent experiments). *G,* unprimed or IFNγ-primed WT L929 were stressed with 100 μM sodium arsenite or 200 mM NaCl for 6 h with or without pretreatment of 10 mM NAC, which is a ROS scavenger, cell viability was assayed using neutral *red* staining. Representative pics of cells were shown, *arrows* indicate dead cells (mean values ± SD; Student's *t* test; ns *p* > 0.1, ∗∗∗∗*p* < 0.0001, n = 2 independent experiments, five replicates per experiment). *H,* immunoblot analysis with indicated antibodies in unprimed or IFNγ-primed WT L929 cells stressed with 100 μM sodium arsenite or 200 mM NaCl for 6 h with or without NAC pretreatment (n = 2 independent experiments). MLKL, mixed lineage kinase domain-like protein.

Contrary to previous findings (29, 30), SA-induced cell death in IFNγ-primed or ZBP1-expressing cells is independent of SGs formation in our model. To identify novel regulators of arsenic-induced, ZBP1-dependent cell death, we performed a genome-wide CRISPR-Cas9 screen in L929 cells. Top-scoring guide RNAs targeted ZBP1, IFNGR2, JAK1, JAK2, RIPK3, and MLKL, validating our screening approach (Fig. 7*A*). Notably, genes involved in SGs formation were not enriched among the recovered hits (Fig. S6*A*).

Intriguingly, four independent sgRNAs targeting KEAP1, a negative regulator of the antioxidant transcription factor NRF2, emerged as one of the top candidates. KEAP1 KO significantly protected cells from arsenic-induced cell death, while KEAP1 reconstitution restored cell death sensitivity (Fig. 7, *B* and *D*). Consistently, immunoblot analysis revealed that in KEAP1 deficient cells, phosphorylation of MLKL was prevented upon IFNγ plus SA treatment (Fig. 7*F*). In contrast, hyperosmotic stress-induced cell death was independent of KEAP1 (Fig. 7*B*). Increased NRF2 expression in KEAP1-deficient cells correlated with reduced cellular reactive oxygen species (ROS) levels upon SA treatment (Fig. 7, *C* and *E*). The antioxidant NAC inhibited both SA- and IFNγ/SA-induced cell death, but not NaCl- or IFNγ/NaCl-induced cell death (Fig. 7, *G* and *H*), consistent with the data obtained in KEAP1 KO cells. These findings were also confirmed in MLKL-deficient cells (Fig. S6, *B*–*H*).

### SLC9A1 contributes to osmotic stress-induced cell death

Our findings indicate that distinct mechanisms of cell death occur in response to arsenic and hyperosmotic stress responses. Unlike arsenic stress, hyperosmotic stress triggers SGs formation independently of G3BP1/2. Moreover, we observed that cell death induced by hyperosmotic stress was unaffected by G3BP1/2 deficiency or the status of KEAP1.

Given the previously established role of SLC9A1 in activating RIPK3 during osmotic stress-induced necroptosis through intracellular pH regulation (31), we investigated its potential involvement in IFNγ-primed, osmotic stress-induced cell death. We utilized ethyl-isopropyl amiloride (EIPA), a specific SLC9A1 inhibitor, to assess this hypothesis. Consistent with previous reports, EIPA pretreatment partially mitigated NaCl-induced cell death (Fig. S7*A*). Importantly, EIPA also exhibited partial inhibition of IFNγ/NaCl-induced cell death (Fig. S7*B*). Consistently, SLC9A1 KO cells displayed partial protection at early time points. However, at later stages, KO cells succumbed to death at levels comparable to WT cells (Fig. S7*C*). These findings suggest that SLC9A1 contributes to early cell death, as previously reported, but becomes dispensable at the later stage when multiple cell death pathways are engaged. In addition, we tested the effect of EIPA in response to SA. Unlike the NaCl condition, where EIPA delayed early cell death, SA-treated cells were not protected by EIPA at either early or late time points (Fig. S7*D*). These findings suggest a potential role for SLC9A1 in the context of IFNγ-primed, osmotic stress-induced cell death.

### ZBP1 drives arsenic-induced tissue injury

**Figure 8:**
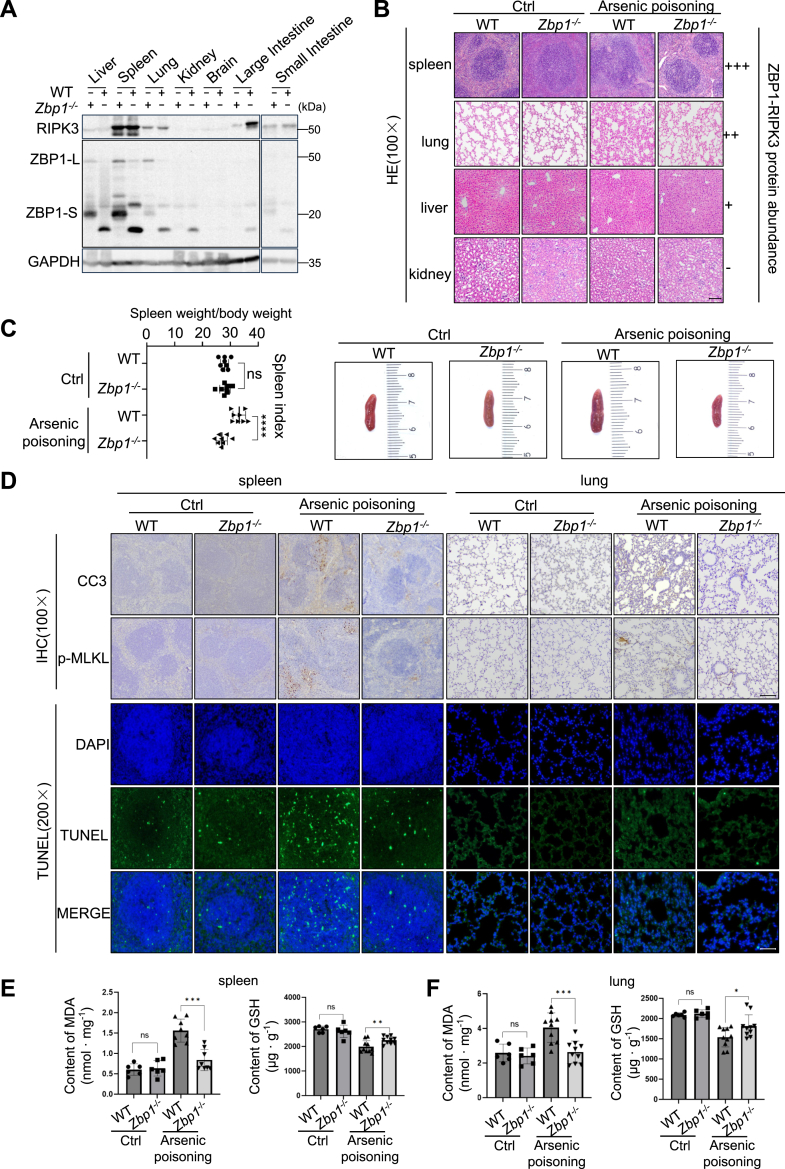
**ZBP1 drives arsenic-induced tissue injury.***A,* immunoblotting analysis of the expression level of RIPK3 and ZBP1 in the liver, spleen, lung, brain, large intestine, and small intestine from WT and *Zbp1*^−/−^ mice. *B,* representative H&E-stained sections of spleen, lung, liver and kidney from WT and *Zbp1*^−/−^ mice supplied with drinking water with or without sodium arsenite (n = 8 mice for each group). *C,* spleen weight index and representative pics of spleens of WT or *Zbp1*^−/−^ mice supplied with drinking water with or without sodium arsenite (mean values ± SD; Student's *t* test; ns *p* > 0.5, ∗∗∗∗*p* < 0.0001, n = 8 mice for each group). *D,* representative images of sections stained for cleaved caspase-3, p-MLKL, DAPI or TUNEL of liver and lung from WT or *Zbp1*^−/−^ mice supplied with drinking water with or without sodium arsenite (n = 3 mice for each group). *E,* the MDA and glutathione levels were assessed to determine the lipid oxidation levels and antioxidant capacity in the spleen of WT and *Zbp1*^−/−^ mice supplied with drinking water with or without sodium arsenite (mean values ± SD; Student's *t* test; ns *p* > 0.05, ∗∗*p* < 0.01, ∗∗∗*p* < 0.001, n = 6 mice for each group). *F,* The MDA and glutathione levels were assessed to determine the lipid oxidation levels and antioxidant capacity in the lung of WT or *Zbp1*^−/−^ mice supplied with drinking water with or without sodium arsenite (mean values ± SD; Student's *t* test; ns *p* > 0.05, ∗*p* < 0.05, ∗∗∗*p* < 0.001, n = 6 mice for each group). MDA, malondialdehyde; MLKL, mixed lineage kinase domain-like protein.

Arsenic, a known human carcinogen, induces severe tissue damage and organ dysfunction (32, 33, 34). Given the complex interplay of cell death pathways in response to arsenic exposure, we investigated the role of ZBP1 in arsenic-induced pathology. RIPK3 expression was highest in the spleen, followed by the large intestine, lung, and small intestine, whereas ZBP1 was moderately expressed in the liver, spleen, and lung (Fig. 8*A*).

To investigate the responses of WT control and ZBP1 KO (35) mice after exposure to arsenic, we supplied sodium arsenite in water to mice for 3 months. This arsenic exposure induced splenomegaly and histological abnormalities in the spleen and lungs of WT mice but not ZBP1-deficient mice (Fig. 8, *B* and *C*). The other tissues such as liver and kidney appear quite normal in WT control animals. These tissue-specific effects correlated with ZBP1 and RIPK3 expression levels. We also observed increased TUNEL, cleaved caspase-3, and p-MLKL staining in the arsenic-exposed spleen, indicative of apoptosis and necroptosis, which were significantly reduced in ZBP1-deficient mice (Fig. 8*D*).

Interestingly, apoptotic and necroptotic cells were found also in locations where immune cells reside. Therefore, we investigated whether the identified signaling pathway is also active in immune cell populations, particularly macrophages. As shown in Figures S8 and S9*A*, fluorescence immunohistochemistry revealed that when WT mice were exposed to sodium arsenite, some F4/80 (macrophage marker)-positive cells were co-localized with cleaved caspase-3 (CC3) or p-MLKL-positive signals in spleen and lung sections. In contrast, ZBP1 KO mice showed no co-localization, indicating that macrophages are indeed affected by ZBP1-dependent cell death in response to sodium arsenite *in vivo*. We further examined the signal transduction events in the macrophage cell line RAW264.7. As demonstrated in Figure S10, *A* and *B*, RAW264.7 cells are susceptible to sodium arsenite-induced cell death in a ZBP1-dependent manner, similar to fibroblasts. Western blot analysis confirmed that macrophages undergo ZBP1-dependent MLKL and caspase activation in response to sodium arsenite treatment (Fig. S10*C*).

Both necroptosis and secondary necrosis at late stage of apoptosis are able to release damage-associated molecular patterns into the extracellular space and trigger inflammation. Excessive inflammatory response further damages the surrounding normal tissues (36, 37). Therefore, we have examined additional markers of tissue damage and inflammation *in vivo*. WT mice exposed to arsenic-containing water showed significantly elevated lactate dehydrogenase (LDH) and alanine aminotransferase (ALT) levels (Fig. S9*B*). LDH serves as a general indicator of tissue damage (38), while ALT is traditionally considered a liver-specific marker. Despite normal liver histopathology in WT mice exposed to sodium arsenate (Fig. 8*B*), we observed elevated ALT levels. However, it is known that ALT can also be released from other tissues, particularly skeletal and cardiac muscle, making it a marker of generalized tissue damage rather than exclusively hepatic injury (39). In the context of sodium arsenate toxicity, the elevated ALT levels in our study may reflect subclinical hepatocellular stress that has not yet progressed to overt histological changes in liver, or alternatively, may indicate extrahepatic tissue damage. Importantly, ZBP1 deficiency significantly reduced these elevated biomarkers, supporting ZBP1's role in mediating arsenic-induced tissue damage *in vivo*. Furthermore, Arsenic-induced oxidative stress, evidenced by elevated malondialdehyde and decreased glutathione levels, was attenuated in spleens and lungs of ZBP1-deficient mice (Fig. 8, *E* and *F*), suggesting that oxidative stress induced ZBP1-dependent pathway plays an important role in tissue damage in mice exposed to arsenic-containing water.

## Discussion

ZBP1, a Z-form nucleic acid sensor, plays crucial roles in infection and inflammation by mediating cell death through interactions with other RHIM-containing molecules (12, 13, 14, 15, 16, 17, 40). However, the mechanisms of ZBP1 activation under various environmental stressors remain unclear. Our study reveals that in ZBP1-expressing cells, arsenic- or hyperosmotic stress initially triggers necroptosis, followed by a shift to apoptosis and pyroptosis, particularly when necroptosis is prevented.

While stressed cells produce distinct SGs, which is protein and RNA aggregates formed under various stress conditions in the cytoplasm, we found them dispensable for arsenic- and hyperosmotic stress-induced cell death. Two studies reported that sodium arsenite-induced SGs are sensed by ZBP1, resulting in necroptosis (29, 30). These studies proposed that arsenite exposure in ZBP1-expressing cells generates ROS, inducing SGs formation and subsequent ZBP1 recruitment to SGs, leading to RIPK3-dependent necroptosis. Our data align with these reports in that sodium arsenite induces ZBP1 and ROS-dependent cell death. However, we did not observe a role of SGs in arsenic stress-dependent cell death. Particularly, we demonstrated that both pharmacologic (emetine/CHX) and genetic (G3BP1/2 KO) approaches to inhibit SGs did not form SGs in response to arsenic stress; the cell death was not prevented. The reason for this discrepancy is currently unclear. Intriguingly, while arsenic stress initially prompts ZBP1-dependent necroptosis, the mode of cell death shifts to apoptosis and pyroptosis at later time points, especially when necroptosis is deficient. This dynamic transition in cell death mechanisms represents a previously unrecognized aspect of stress response. This novel connection between ZBP1 and the cellular stress response machinery provides a new perspective on environmental stress-induced cell death mechanisms.

Our genome-wide CRISPR-Cas9 screen further supports the notion that SGs formation is not a major driver of arsenite-dependent cell death, as genes involved in SGs formation were not enriched in the screening. Instead, KEAP1, a negative regulator of the antioxidant transcription factor NRF2, emerged as a top candidate involved in arsenite-dependent ZBP1-mediated cell death, reinforcing the importance of ROS in this process. The diverse proposed functions of SGs, with conflicting experimental data, have hindered the development of a comprehensive SGs model (41). The role of SGs in cell death also remains a contentious topic. While some studies suggest that SGs protect cells from death (42, 43, 44, 45), others, including two recent reports (29, 30), propose that SGs instead promote cell death. Our findings indicate that SGs play no role in arsenic stress-induced cell death. In our experiments, we inhibited SGs formation using CHX or emetine. These inhibitors effectively prevented sodium arsenite-induced SGs formation but did not protect cells from death (Fig. 6, *C* and *D*). Moreover, we demonstrated that although G3BP1/2-deficient cells did not form SGs in response to arsenic stress, the cell death was not prevented (Fig. 6, *F* and *G*). Despite these conflicting views on the role of SGs, there is a consensus across all studies; including ours that environmental stressors can promote ROS generation in cells and this ROS production appears to be a crucial factor in determining cell fate. Arsenic, an environmental pollutant present in soil, air, food, and water, is linked to various adverse health outcomes upon chronic exposure, including cancer, lung damage and inflammation (32, 33, 34). Our *in vivo* studies reveal that chronic arsenic exposure in WT mice results in disrupted tissue structure, elevated cleaved caspase-3, and MLKL activation. These effects are significantly reduced in *Zbp1* deficient mice.

We also found that hyperosmotic stress triggers ZBP1-dependent cell death. However, the mechanisms underlying cell death in response to arsenic and hyperosmotic stress were distinct from each other. Unlike arsenic stress, hyperosmotic stress induced SGs formation independently of G3BP1/2. Moreover, hyperosmotic stress-induced cell death was unaffected by G3BP1/2 deficiency or KEAP1 status, indicating a different mechanistic pathway. Given that SLC9A1 has been previously reported to activate RIPK3 during osmotic stress-induced necroptosis, we investigated its potential involvement in ZBP1-dependent osmotic stress-induced cell death. Treatment with EIPA (an SLC9A1 inhibitor) or SLC9A1 KO provided partial protection against ZBP1-dependent hyperosmotic stress-induced cell death, suggesting that SLC9A1 contributes to this alternative cell death pathway. These findings demonstrate that while ZBP1 serves as a common mediator, the downstream signaling mechanisms differ depending on the type of cellular stress encountered.

## Experimental procedures

### Cell lines and cell culture

The L929 and RAW264.7 cell lines were gifts from Professor J.Han’s lab. MEFs cells were obtained through immortalization of primary cells. KO cell lines were generated by using CRISPR/Cas9 method. The disruption of target gene was determined by the sequencing of gene loci and by the immunoblotting of cell lysates with antibodies. Cells were cultured using high-glucose DMEM (Gibco, C11995500BT) with 10% fetal bovine serum (ABW, AB-FBS-1050), 1× L-glutamine (Solarbio, g0200), 1× sodium pyruvate (Solarbio, SP0100), and 1× penicillin-streptomycin (NCM, C100C5). All cell lines were cultured at 37 °C with 5% CO_2_. Following drug were used in this paper to treat cells: Recombinant Mouse IFN-gamma Protein (ABclonal, RP01070), Sodium arsenite (AIKE REAGENT, 101,486), Sodium Chloride (Sangon, A610476), Z-VAD-FMK (MCE, HY-16658B), Cycloheximide (MCE, HY-12320), Emetine dihydrochloride hydrate (MACKLIN, E877712), NAC (TargerMOI, T0875), EIPA (TargetMOI, TQ0157).

### Cell death assays

Cell death was analyzed by using neutral red staining. Briefly, 2 × 10^4^ cells were seeded in 96-well plates with white walls (JET, TCP010096). After 12 h, the cells were treated with reagents for the indicated. To measure cell death, 4 μg ml^−1^ neutral red was added in DMEM supplemented with 10% FBS for 1 h. The cells were then washed twice with 1× PBS to remove any unabsorbed neutral red. Following that, 100 μl solution containing 50% absolute ethanol and 10% glacial acetic acid was added to each well to release the neutral red absorbed by viable cells. After standing for 1 h, the absorbance at 540 nm was measured using a microplate reader (TECAN, Infinite F50).

### Propidium iodide staining

After the cells are given the specified stimulus, they are stained with 10 μg ml^−1^ Propidium iodide (Beyotime, ST511), and photographed under a microscope 30 min later.

### Evaluation of mitochondrial damage

After the cells are given the specified stimulus, they were stained with 10 μM JC-1 (TargerMOI T15609) staining solution at 37 °C in the dark for 30 min, followed by washing twice with 1× PBS. Stained sections were observed and photographed using a Zeiss inverted confocal microscope (LSM 880).

### Immunoblotting

Cell lysates were prepared by direct cell lysis in 1× loading buffer (2% SDS, 10% glycerol, 0.02% bromophenol blue, 50 mM Tris-HCl pH 7.8). Lysates were separated by SDS–PAGE, transferred to Immobilon-P PVDF membranes (Millipore, IPVH00010) and analyzed by immunoblotting with indicated antibodies. Secondary horseradish peroxidase (HRP)-coupled antibodies against rat, rabbit or mouse were used to detect proteins using chemiluminescence with ECL (Vazyme, E411-05), and signal was measured with a chemiluminescence imaging system (BIO-OI, OI900). Antibodies used for immunoblotting: MLKL (Millipore, MABC604), p-MLKL (CST, 37333S), p-MLKL (Abcam, ab196436), ZBP1 (AdipoGen, AG-20B-0010-C100), GAPDH (Abclonal, AC033), β-Actin (Abclonal, AC026), RIPK3 (CST, 15828s), CASP1 (AdipoGen, AG-20B-0042-C100), CASP3 (CST, 9662S), Cl-CASP8 (CST, 8592T), FL-CASP8 (ABclonal, A11324), CASP9 (Abcam, ab202068), GSDMD (Abcam, ab219800), GSDME (Abcam, ab215191), RIPK1 (CST, 3493S), G3BP1 (Proteintech, 13057-2-AP), G3BP2 (CST, 31799s), KEAP1 (CST, 8047S), NRF2 (Abcam, ab137550), TIA1 (Proteintech, 12133-2-AP), SLC9A1 (Proteintech, 29761-1-AP), HRP-conjugated Goat anti-Mouse IgG (Abclonal, AS003), HRP-conjugated Goat anti-Rabbit IgG (Abclonal, AS014), HRP-conjugated Goat anti-Rat IgG (Abclonal, AS028). The antibodies against CASP1, GSDMD, GSDME, NRF2, and TIA1 have been validated *via* KO or knockdown experiments in other studies, while the other antibodies used in this study have been validated through KO experiments.

### Lentivirus preparation and infection

A total of 293T cells were cotransfected with plasmid constructs and lentiviral packaging plasmids (psPAX2 and pMD2.G) by PEI transfection. Fresh medium was replaced after 12 h. The lentivirus-containing supernatant was collected 36 h later and used for infection with 10 μg ml^−1^ polybrene. Plates were centrifuged at 2500×rpm for 30 min at 37 °C and returned to the cell incubator. The infectious medium was changed 12 h later.

### Co-immunoprecipitation

After pretreating with 10 ng ml^−1^ IFNγ (ABclonal RP01070) 24 h and stressing the cells with 100 μM sodium arsenite (AIKE REAGENT 101486) 3 h, WT and MLKL-deficient L929 were collected in ice-cold 1x PBS and resuspended in lysis buffer. The resuspended cell pellets were sonicated and centrifuged at 18,000*g* for 15 min at 4 °C. The supernatants were collected for Immunoblotting or Immunoprecipitation. For immunoprecipitation of endogenous protein, the Protein A/G Magnetic Beads were coupled with antibody ZBP1 (AdipoGen AG-20B-0010-C100) at 4 °C for 2 h. Then cell lysates were incubated with antibody-coupled beads overnight at 4 °C. The next day, the beads were washed with lysis buffer for three times and the immunoprecipitates were eluted off the beads with 1.2x loading buffer (2.4% SDS, 12% glycerol, 0.024% bromophenol blue, 60 mM Tris-HCl pH 7.8) for 10 min at 100 °C and subjected to immunoblotting. To minimize the influence of IgG and heavy chain in immunoblotting, the Mouse IgG control (Proteintech, B900620) and anti-Mouse IgG light chain (ABclonal AS061) were used.

### CRISPR screening

The CRISPR KO library was purchased from GENEWIZ, Inc. in Suzhou. Amplification of the library and preparation of the lentivirus were performed according to the manufacturer’s instructions. WT L929 was infected with the gRNA lentivirus library with a multiplicity of infection of 0.3. 48 h after infection, and cells were reseeded in fresh medium supplemented with puromycin (10 mg ml^−1^) to eliminate noninfected cells. The experimental group was pretreated with 10 ng ml^−1^ interferon-γ for 24 h, followed by stimulation with 100 μM sodium arsenite for 4 h. After three rounds of screening, more than 5 × 10^5^ cells from the control and experimental groups were collected. The high-throughput sequencing and analysis was completed by GENEWIZ, Inc.

### ROS assay

H2DCFDA staining was performed to detect the level of ROS in cells. 2.5 × 10^5^ cells were seeded on 12-well plate and treated with indicated reagents, and then incubated with pre-warmed 5 μM H2DCFDA in DMEM supplemented with 10% FBS for 30 min at 37 °C. Then, the cells were resuspended in 1 × PBS and the fluorescence intensity of 2′, 7′-dichlorofluorescein was evaluated using flow cytometry (Beckman CytoFlex S). The data was analyzed using Flowjo software (https://www.flowjo.com/).

### Mice

*Zbp1*^−/−^ mice (LMSRC-00017) were from Xiamen University Laboratory Mouse Shared Resource Center (35). All mice were average initial weight 20 g and 6 to 8 weeks of age and maintained in specific-pathogen-free condition. All experimental procedures were conducted in strict accordance with the guidelines outlined by the Institutional Animal Care and Use Committee of the Laboratory Animal Center at Xiamen University. The protocols for animal use were thoroughly reviewed and approved by the Animal Ethical and Welfare Committee of the Laboratory Animal Center of Xiamen University (approval no. XMULAC20220149).

### Establishment of an arsenic poisoning mouse model

*Zbp1*^−/−^ and WT littermate control animals were supplied drinking water with or without 50 mg l^−1^ of sodium arsenite for 3 months. Before sacrifice, the mice were fasted for 12 h. Mouse body weights were measured and recorded. The mice were anaesthetised with tribromoethanol and euthanized by cervical dislocation. Liver, spleen, lung, kidney, brain, large intestine and small intestine of mice were collected after dissection. These tissues were partly fixed with 4% paraformaldehyde and partly stored in a freezer at −80 °C for subsequent protein detection and analysis.

### Spleen coefficient calculation

After dissection, the spleen weight of the mouse was measured, and the spleen coefficient was calculated using the following formula: Spleen Coefficient = (Spleen Weight/Body Weight) × 100%.

### Measurement of oxidative stress markers in the spleen and lung tissues of mice

Mouse spleen and lung tissues were taken and homogenized using a high-speed low-temperature tissue grinder. Level of malondialdehyde was measured using the thiobarbituric acid method with the malondialdehyde content detection kit (Solarbio, BC0025), and the level of reduced glutathione was determined using a microplate method with reduced glutathione test kit (Solarbio, BC1175). Experiments were performed strictly according to the instructions of the corresponding kits.

### HE staining (hematoxylin and eosin staining)

The liver, spleen, lung, and kidney tissue samples were fixed in 4% paraformaldehyde, followed by routine dehydration, wax immersion, embedding, and slicing into 4 μm sections. HE staining was performed using a commercial kit (Solarbio G1120-3), and histopathological changes were observed under an optical microscope at 100× magnification.

### Immunohistochemistry

For immunohistochemical analysis, slides were rehydrated and incubated with peroxidase blocking buffer (ZSGB-BIO, PV-9000). Slides were washed and antigen retrieval was performed in 0.01 M citrate buffer (Servicebio, G0002-2L) in a microwave oven. Sections were blocked in 4% FCS with Avidin/Biotin Blocking Kit (Biozol, VEC-SP-2001) for 30 min. Primary antibodies anti-CC3 (CST, 9661S) and anti-p-MLKL (Abcam, ab196436) were incubated with samples overnight at 4 °C. Fluorence-labeled Secondary antibody and DAB were provided by kit (Biozol, VEC-PK-6100). After counterstaining with hematoxylin sections were mounted with Entellon (MERCK) mounting medium. Histological sections were observed and photographed with a microscope (OLYMPUS, CKX53).

### Immunofluorescence

Twelve hours after seeding 1 × 10^5^ cells on a coverslipcells were treated with indicated drugs. The cells were fixed with 4% paraformaldehyde in PBS solution at room temperature and permeabilized by 0.3% Triton in PBS. After treating blocking solution (Beyotime, P0102, the cells were treated with anti-ZBP1 (AdipoGen, AG-20B-0010-C100), anti-G3BP1 (Proteintech, 13057-2-AP), or anti-TIA1 (Proteintech, 12133-2-AP) and incubated overnight at 4 °C. The staining was visualized by coraLite594-conjugated goat anti-mouse IgG (Proteintech, SA00013-3) or coraLite488-conjugated goat anti-rabbit IgG (Proteintech, SA00013-2. Images were obtained using a Zeiss inverted confocal microscope (LSM 880).

### TUNEL assay

Sections (4 μm) were rehydrated and permeabilized with proteinase K working solution. Experiments were conducted according to the operating instructions of the TUNEL Apoptosis Detection Kit (Vazyme, A112-03). After coverslipping, stained sections were observed and photographed under a fluorescence microscope.

### Multiplex immunohistochemical

The collected mouse spleen and lung tissue sections were dewaxed with conventional xylene and hydrated with gradient alcohol. The multiplex immunohistochemical staining of the tissue sections was performed using the Double-labeling Three-color Multiple Fluorescence Staining Kit (AFIHC033, Aifang Biologcal). According to the manufacturer's product instructions, the antigens were exposed by microwave repair. The sections were incubated with 3% hydrogen peroxide solution at room temperature for 15 min and then dropped with 10% goat serum for blocking for 15 min. The primary antibody CC3 or p-MLKL was added and incubated overnight at 4 °C, and then washed 3 times with Phosphate Buffered Saline with Tween-20 (PBST). Polymer-HRP anti-mouse/rabbit universal secondary antibody IgG (AFIHC001, Aifang Biotechnology) was dropped and incubated at room temperature for 30 min. Subsequently, the sections were washed with PBST, and TYR fluorescent dye was dropped for reaction for 10 min, followed by washing with PBST for 3 times. The CC3 or p-MLKL antibody was removed by microwave, and then the sections were washed with PBST for 3 times. After dropping goat serum for blocking, the primary antibody F4/80 was added, and the above operations were repeated until the color development was completed. Anti-fluorescence quenching mounting medium (containing DAPI) was dropped for mounting, and a three-channel fluorescence digital slide scanner (model Leica Aperio Versa 200) was used to collect images of the multiplex immunohistochemical slides.

### Serological experiments

Centrifuge the blood of mice to extract serum. LDH and aspartate aminotransferase detection kits (Shanghai Acmec Biochemical Technology Co., Ltd) were used to evaluate the degree of inflammation in mice. The optical density was measured according to the manufacturer's instructions.

### Generation of KO cell lines

To ensure the KO effect, at least two sgRNAs were designed through the website https://portals.broadinstitute.org/gppx/crispick/public. Then, the constructed CRISPR-v2 plasmid is mixed with pSPAX2 and PMD2.G at a ratio of 4:3:1 for lentivirus packaging. The packaged virus is collected through a 0.45 μm filter and then used to infect the target cells. When the cell density reaches 50%, the virus is mixed with cell culture medium at a 1:1 ratio for infection. Polybrene is added to a final concentration of 10 μg ml^−1^. The mixture is centrifuged at 2500 rpm at 37 °C for 30 min. After continued culture for 36 h, the infected cells are selected with 10 μg ml^−1^ of puromycin for 48 h. After that, cells were lysed and proteins were collected for Western blot analysis. DNA fragments near the targeted KO site were amplified through PCR for sequencing to verify the KO effect. Single clone cells were selected and expanded to obtain a completely KO clones.

### SgRNA

The sgRNA sequences used in this article are as follows:

**Table:** 

Gene	sgRNA sequence
*Zbp1*	GAAGATCTACCACTCACGTC
	CGAGGCTCATCTCGTTGTGG
	CCACGAGGCTCATCTCGTTG
	CAGGTGTTGAGCGATGACGG
*Ripk3*	GGATCCAGCAGAATGTTAGA
	CGGACACGAAGTCCCACTGG
*Mlkl*	TGCTGCTTCAGGTTTATCAT
	GCACACGGTTTCCTAGACGC
*G3bp1*	GGAGAAGCCTAGTCCCCTGC
	GCAGTCTACGGGCAGAAGGT
	AGGCCCCGGACATGTTGCAC
	CTAGTCCCCTGCTGGTCGGG
*G3bp2*	ATCCACTCCACCATGAACAT
	AAGCTCCCGAGTATTTGCAC
	CCGCCCCACAAGCAGCGGAC
	GGATGCCAGTGGAAAGCCCC
*Ripk1*	AGAAGAAGGGAACTATTCGC
	GGGTCTTTAGCACGTGCATC
*Keap1*	AGCGTGCCACGCAACCGCAT
*Slc9a1*	GGGGCGCATCACTACTCCTG
	GAAGGTCCAGTTCCACTGGT
*Casp3*	CATGCAGAAAGACCATACAT
	AATGTCATCTCGCTCTGGTA
*Casp8*	CTCAGAAGAAGTGAGCGAGT
	AGTCTAGGAAGTTGACCAGC
*Casp9*	GCTGCAAGTCGCGGAGCTCT
	AACTTGAGCACCGATTCCGC

### Quantification and statistical analysis

Data shown in graphs are the mean or mean ± SD. All statistical analyses were conducted using Student's *t* test. All statistical analyses were performed with GraphPad Prism 9 (https://www.graphpad.com/scientific-software/prism/) ns *p* > 0.05, ∗*p* < 0.05; ∗∗*p* < 0.01; ∗∗∗*p* < 0.001; ∗∗∗∗*p* < 0.0001 for all figures. No data were excluded.

## Data availability

The original contributions presented in this study are included in the article/supplementary material. Further inquiries can be directed to the corresponding author.

## Supporting information

This article contains supporting information.

## Conflict of interest

The authors declare that they have no conflicts of interest with the contents of this article.
