# Supplementary material for: ZBP1 orchestrates dynamic transitions between cell death pathways in response to arsenic and hyperosmotic stress
Source: J Biol Chem. 2025 Oct 22;301(12):110856. doi: 10.1016/j.jbc.2025.110856 (PMC12666829; doi:10.1016/j.jbc.2025.110856)
Supplement: Supporting information [file mmc1.pdf]

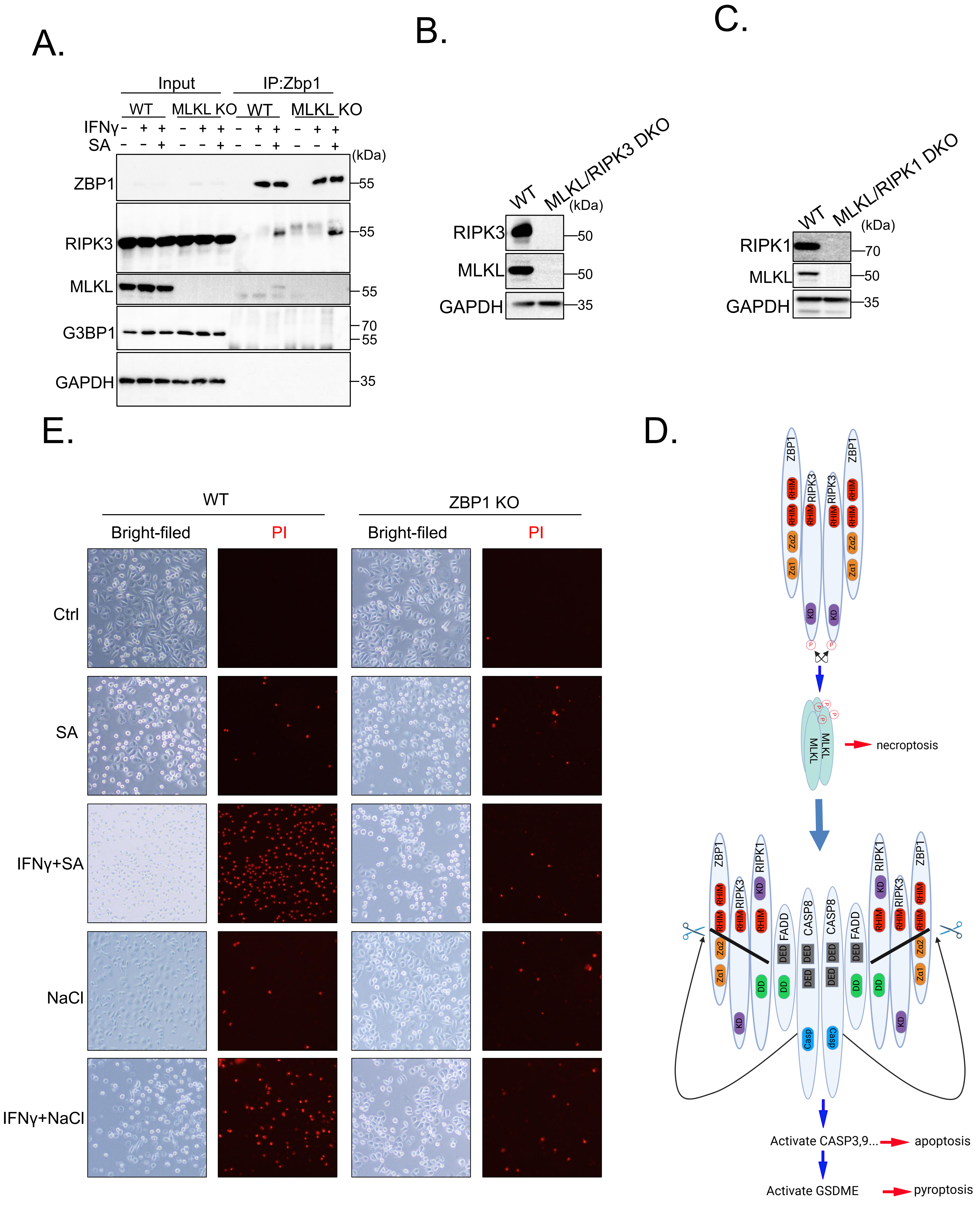
A.
Input
IP:Zbp1
WT
MLKL KO
WT
MLKL KO
IFNγ
SA
(kDa)
ZBP1
RIPK3
MLKL
G3BP1
GAPDH
55
55
55
70
55
35
B.
WT
MLKL/RIPK3 DKO
(kDa)
RIPK3
MLKL
GAPDH
50
50
35
C.
WT
MLKL/RIPK1 DKO
(kDa)
RIPK1
MLKL
GAPDH
70
50
35
D.
ZBP1
RIPK3
RHIM
Zα2
Zα1
KD
MLKL
necroptosis
RIPK1
FADD
CASP8
DED
DD
Casp
Activate CASP3,9...
apoptosis
Activate GSDME
pyroptosis
E.
WT
ZBP1 KO
Bright-filed
PI
Ctrl
SA
IFNγ+SA
NaCl
IFNγ+NaCl

S1

Figure S1.

(A) Immunoblot analysis with the indicated antibodies of ZBP1 immunoprecipitates and total lysates from WT and MLKL KO L929 cells stressed with 100 μM sodium arsenite for 3 hours with or without 10 ng ml^{-1} IFNγ pretreatment (n = 2 independent experiments).

(B) Immunoblot analysis of RIPK3 and MLKL in WT or MLKL/RIPK3 DKO L929 cells.

(C) Immunoblot analysis of RIPK1 and MLKL in WT or MLKL/RIPK1 DKO L929 cells.

(D) Schematic diagram: Under certain stress conditions such as arsenic and osmotic stress, ZBP1 mediates RIPK3/MLKL dependent necroptosis, particularly at early time point. Inhibition of necroptosis, such as through MLKL ablation, promotes apoptosis and pyroptosis signaling pathways controlled by both RIPK3 and RIPK1.

(E) Microscopic analysis of WT or ZBP1 KO L929 after propidium iodide (PI) staining for cell death induced by stress of 100 μM sodium arsenite or 200 mM NaCl for 4 hours with or without IFNγ pretreatment. Bar, 50 μm (n = 3 independent experiments).

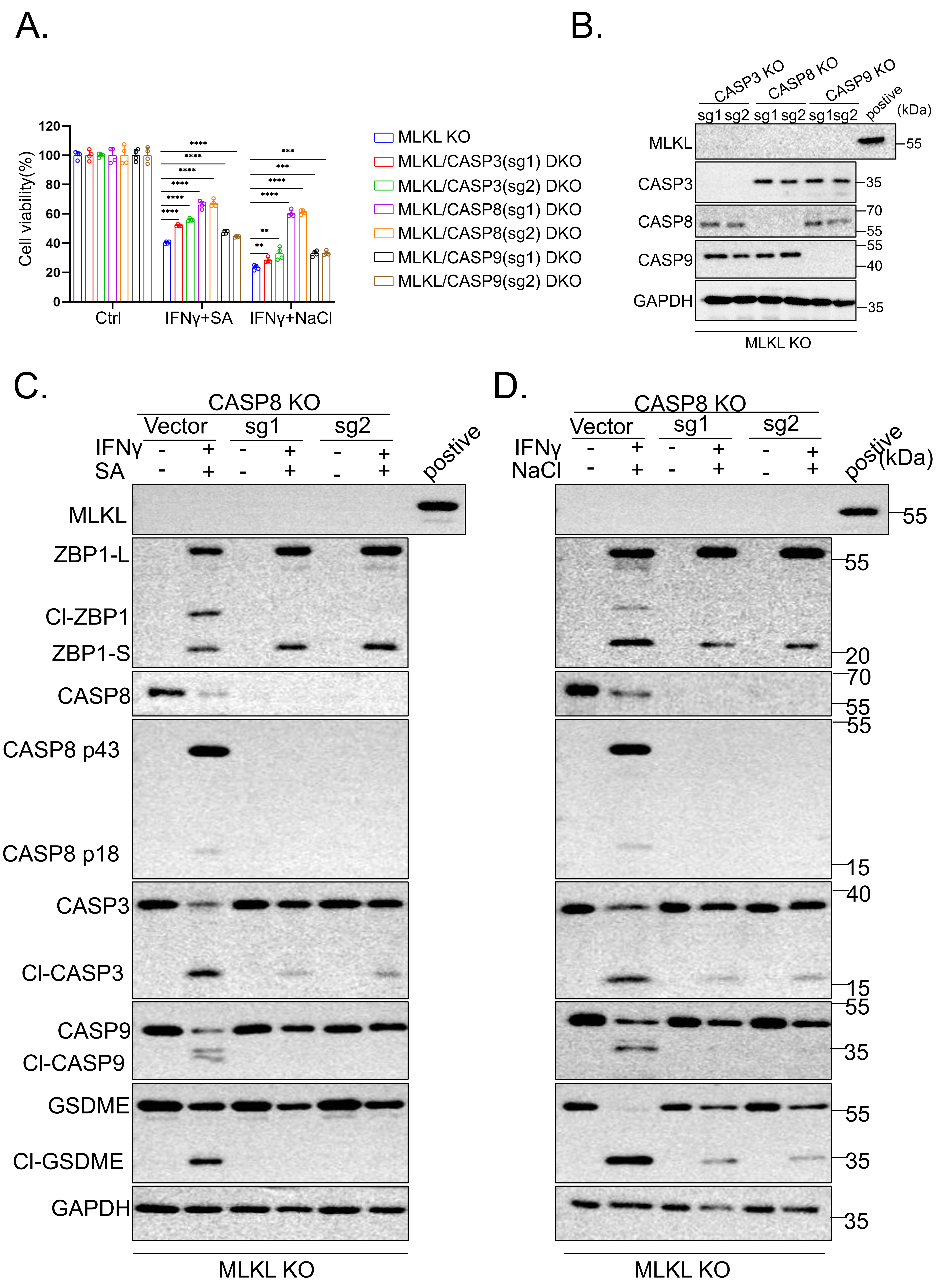
A.
Cell viability(%)
120
100
80
60
40
20
0
Ctrl
IFNγ+SA
IFNγ+NaCl
MLKL KO
MLKL/CASP3(sg1) DKO
MLKL/CASP3(sg2) DKO
MLKL/CASP8(sg1) DKO
MLKL/CASP8(sg2) DKO
MLKL/CASP9(sg1) DKO
MLKL/CASP9(sg2) DKO
B.
CASP3 KO
CASP8 KO
CASP9 KO
sg1 sg2 sg1 sg2 sg1sg2
postive
(kDa)
MLKL
CASP3
CASP8
CASP9
GAPDH
MLKL KO
C.
CASP8 KO
Vector
sg1
sg2
IFNγ
SA
postive
MLKL
ZBP1-L
Cl-ZBP1
ZBP1-S
CASP8
CASP8 p43
CASP8 p18
CASP3
Cl-CASP3
CASP9
Cl-CASP9
GSDME
Cl-GSDME
GAPDH
MLKL KO
D.
CASP8 KO
Vector
sg1
sg2
IFNγ
NaCl
postive
(kDa)
MLKL KO

S2

Figure S2.

(A) MLKL KO, MLKL/CASP3 DKO, MLKL/CASP8 DKO and MLKL/CASP9 DKO L929 were treated with 100 μM sodium arsenite or 200 mM NaCl for 12 hours with IFNγ pretreatment. Cell viability was determined by neutral red staining (mean values ± SD; Student's t-test; **$p < 0.01$; ***$p < 0.001$; ****$p < 0.0001$, n = 2 independent experiments, four replicates per experiment).

(B) Immunoblot analysis of MLKL, CASP3, CASP8 and CASP9 in WT, MLKL KO, MLKL/CASP3 DKO, MLKL/CASP8 DKO and MLKL/CASP9 DKO L929.

(C) Immunoblot analysis of with indicated antibodies in MLKL KO, MLKL/CASP3 DKO, MLKL/CASP8 DKO and MLKL/CASP9 DKO L929 that were treated with 100 μM sodium arsenite or 200 mM NaCl with IFNγ pretreatment (n = 2 independent experiments).

A.

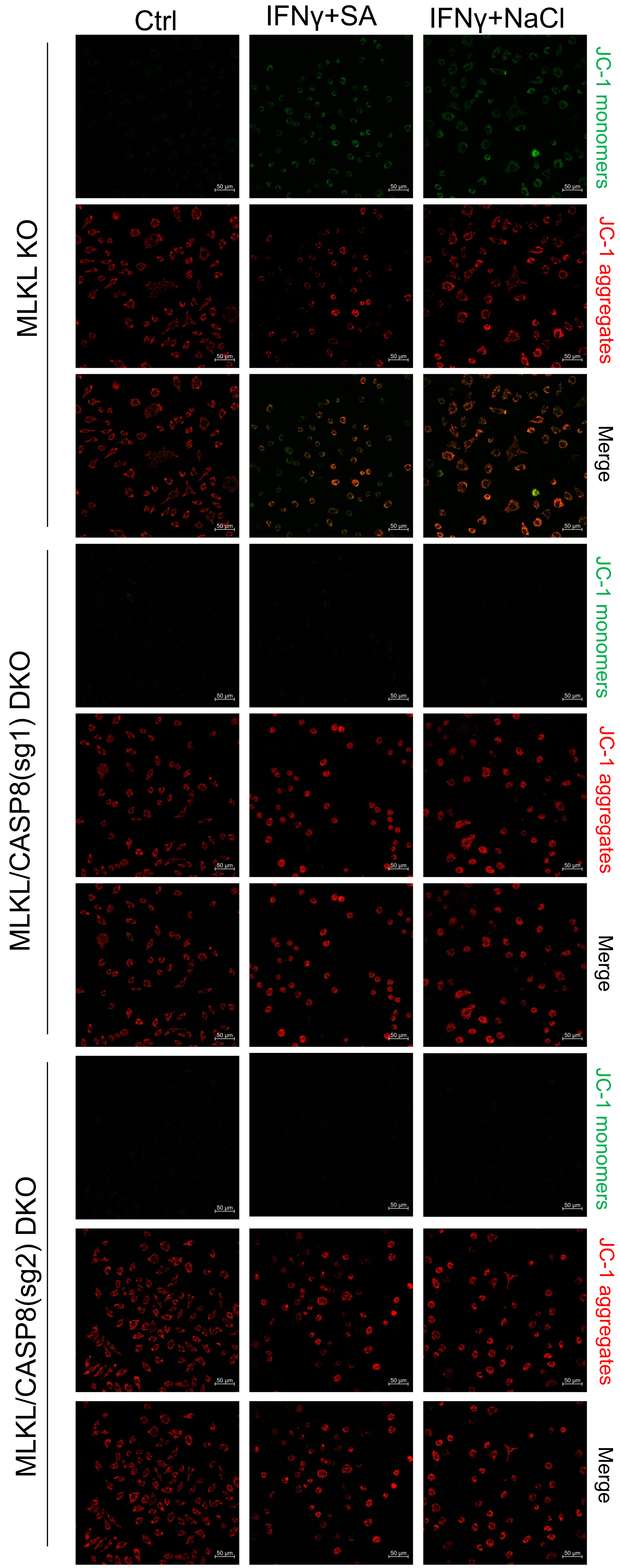

Ctrl
IFNγ+SA
IFNγ+NaCl
MLKL KO
MLKL/CASP8(sg1) DKO
MLKL/CASP8(sg2) DKO
JC-1 monomers
JC-1 aggregates
Merge
50 μm

S3

Figure S3.

(A) JC-1 staining is used to evaluate mitochondrial damage in MLKL KO and MLKL/CASP8 DKO L929 that were treated with 100 μM sodium arsenite or 200 mM NaCl with IFNγ pretreatment. Following mitochondrial damage, JC-1 transitions from aggregates to monomers, accompanied by a shift from red fluorescence to green fluorescence (n = 2 independent experiments).

A.

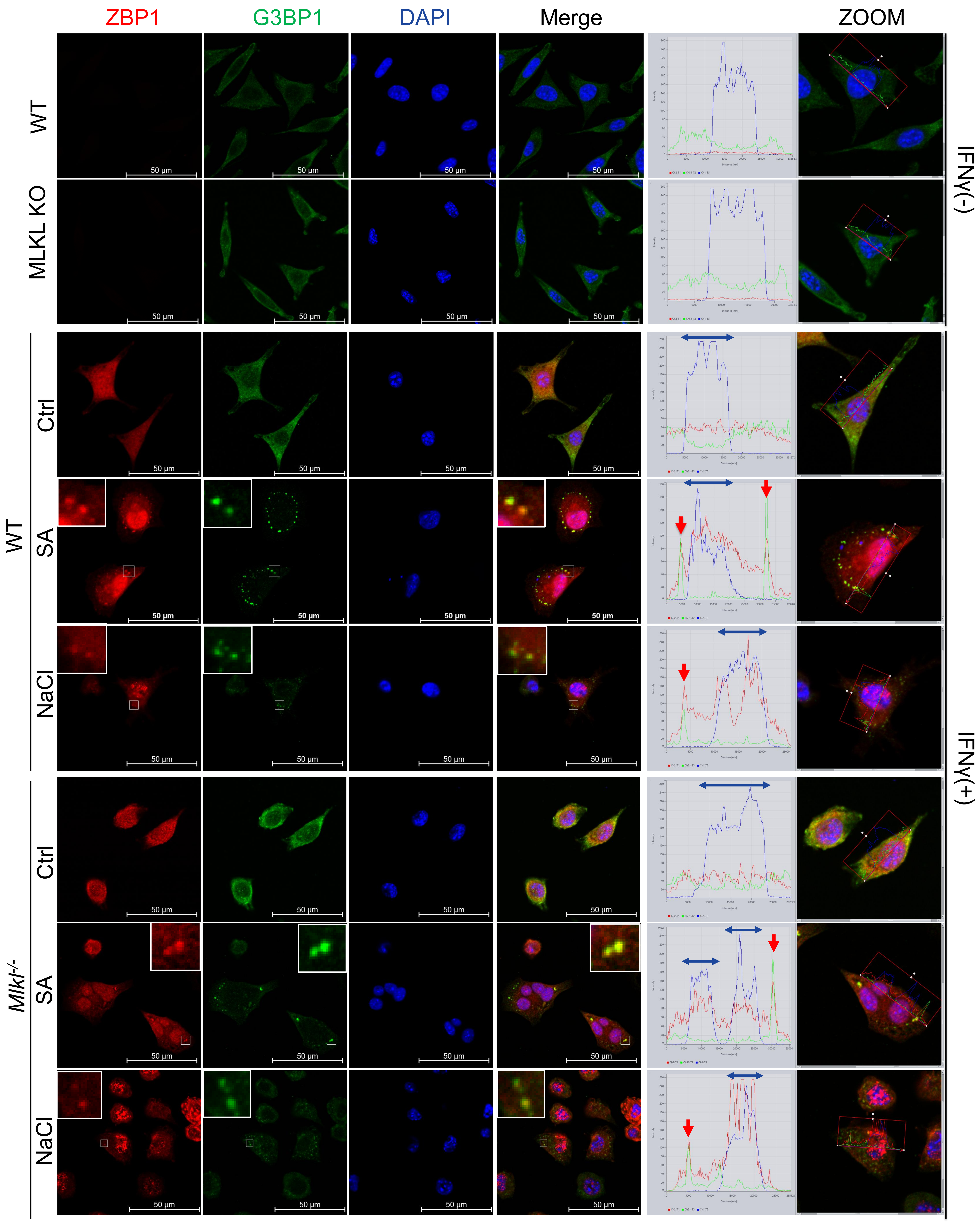

Figure S4.

(A) Representative images of IFNγ primed WT and MLKL KO cells stressed with 100 µM sodium arsenite or 200 mM NaCl stained with ZBP1, G3BP1 and DAPI, Bar, 50 µm (n = 3 independent experiments).

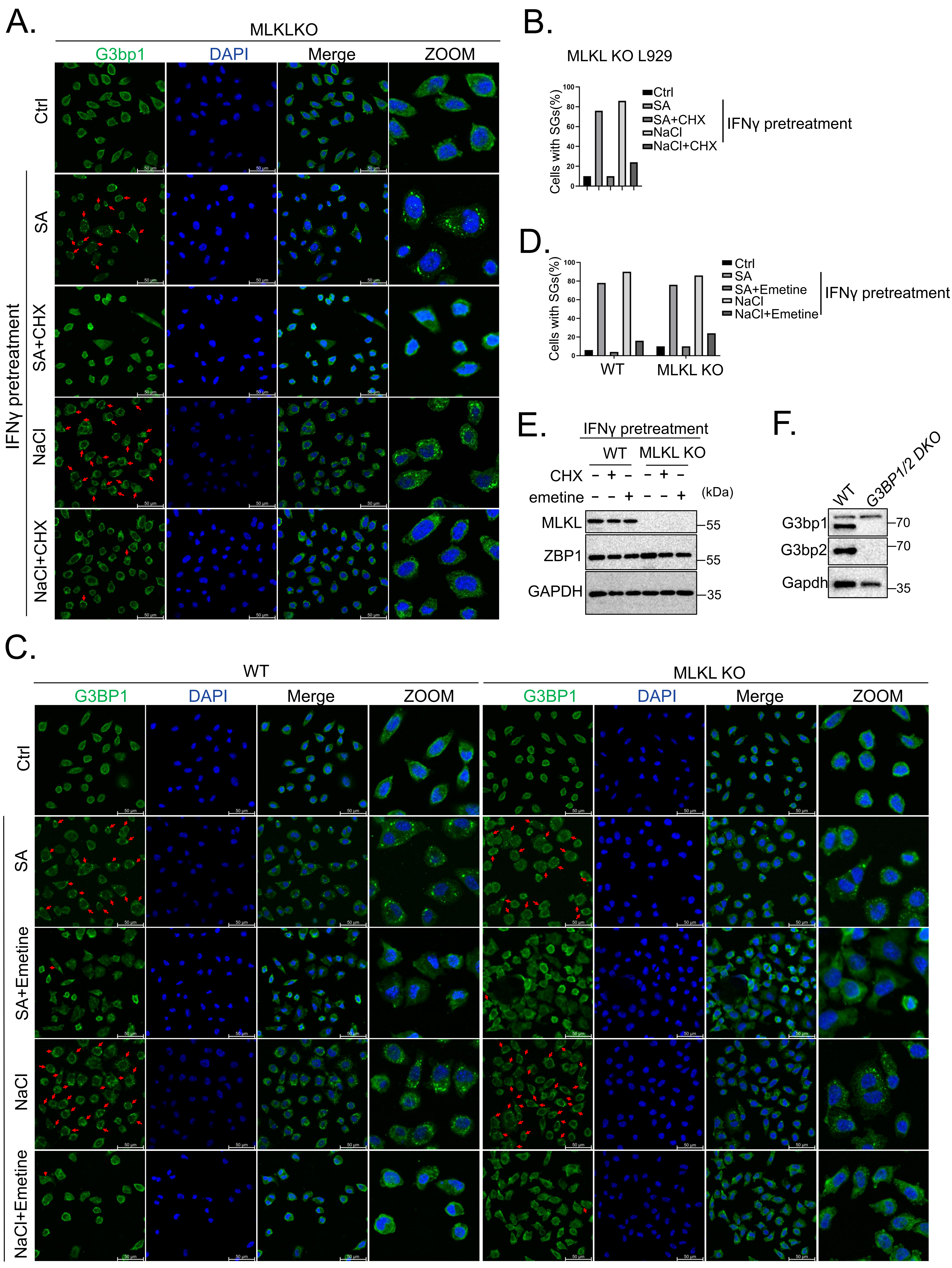
A.
MLKLKO
G3bp1
DAPI
Merge
ZOOM
Ctrl
SA
SA+CHX
NaCl
NaCl+CHX
IFNγ pretreatment
B.
MLKL KO L929
Cells with SGs(%)
Ctrl
SA
SA+CHX
NaCl
NaCl+CHX
IFNγ pretreatment
D.
Cells with SGs(%)
WT
MLKL KO
Ctrl
SA
SA+Emetine
NaCl
NaCl+Emetine
IFNγ pretreatment
E.
IFNγ pretreatment
WT
MLKL KO
CHX
emetine
(kDa)
MLKL
ZBP1
GAPDH
55
55
35
F.
WT
G3BP1/2 DKO
G3bp1
G3bp2
Gapdh
70
70
35
C.
WT
MLKL KO
G3BP1
DAPI
Merge
ZOOM
Ctrl
SA
SA+Emetine
NaCl
NaCl+Emetine
IFNγ pretreatment

S5

Figure S5.

(A-B) Representative images of cells stained with DAPI or immunostained for G3BP1 and graphs depicting quantification of the proportion of G3BP1$^+$ cells in IFNγ primed MLKL KO L929 cells stressed with 100 μM sodium arsenite or 200 mM NaCl for 2 hours with or without 50 μg ml^{-1} CHX pretreatment. Arrows indicate cells that from G3BP1 aggregation, which is a characterization of stress granules (SGs) formation. Bar, 50 μm (n = 2 independent experiments).

(C-D) Representative images of cells stained with DAPI or immunostained for G3BP1 and graphs depicting quantification of the proportion of G3BP1$^+$ cells in IFNγ primed MLKL KO L929 cells stressed with 100 μM sodium arsenite or 200 mM NaCl for 2 hours with or without 20 μg ml^{-1} Emetine pretreatment. Arrows indicate cells that from G3BP1 aggregation, which is a characterization of stress granules (SGs) formation. Bar, 50 μm (n = 2 independent experiments).

(E) Immunoblot analysis of with indicated antibodies in IFNγ primed WT and MLKL KO L929 that were treated with or without 50 μg ml^{-1} CHX or 20 μg ml^{-1} Emetine.

(F) Immunoblot analysis of G3BP1 and G3BP2 in WT or G3BP1/2/ DKO L929 cells.

A.

Gene id	pos|rank	pos|lfc
G3bp1	10420	-0.6737
G3bp2	2151	0.72906
Tia1	17853	-0.30667
Hdac6	3832	0.25087
Prrc2c	12261	-0.1784
Csde1	13793	-0.79986
Ubap2l	16499	-0.031049

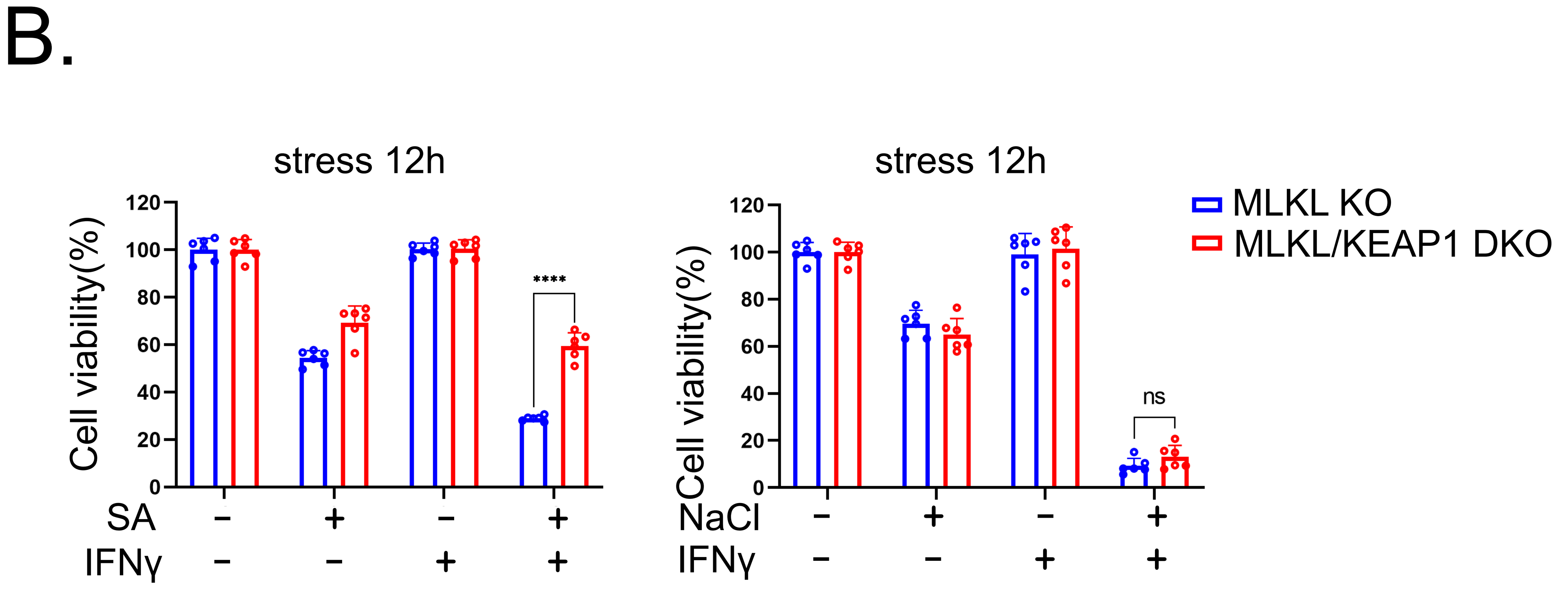

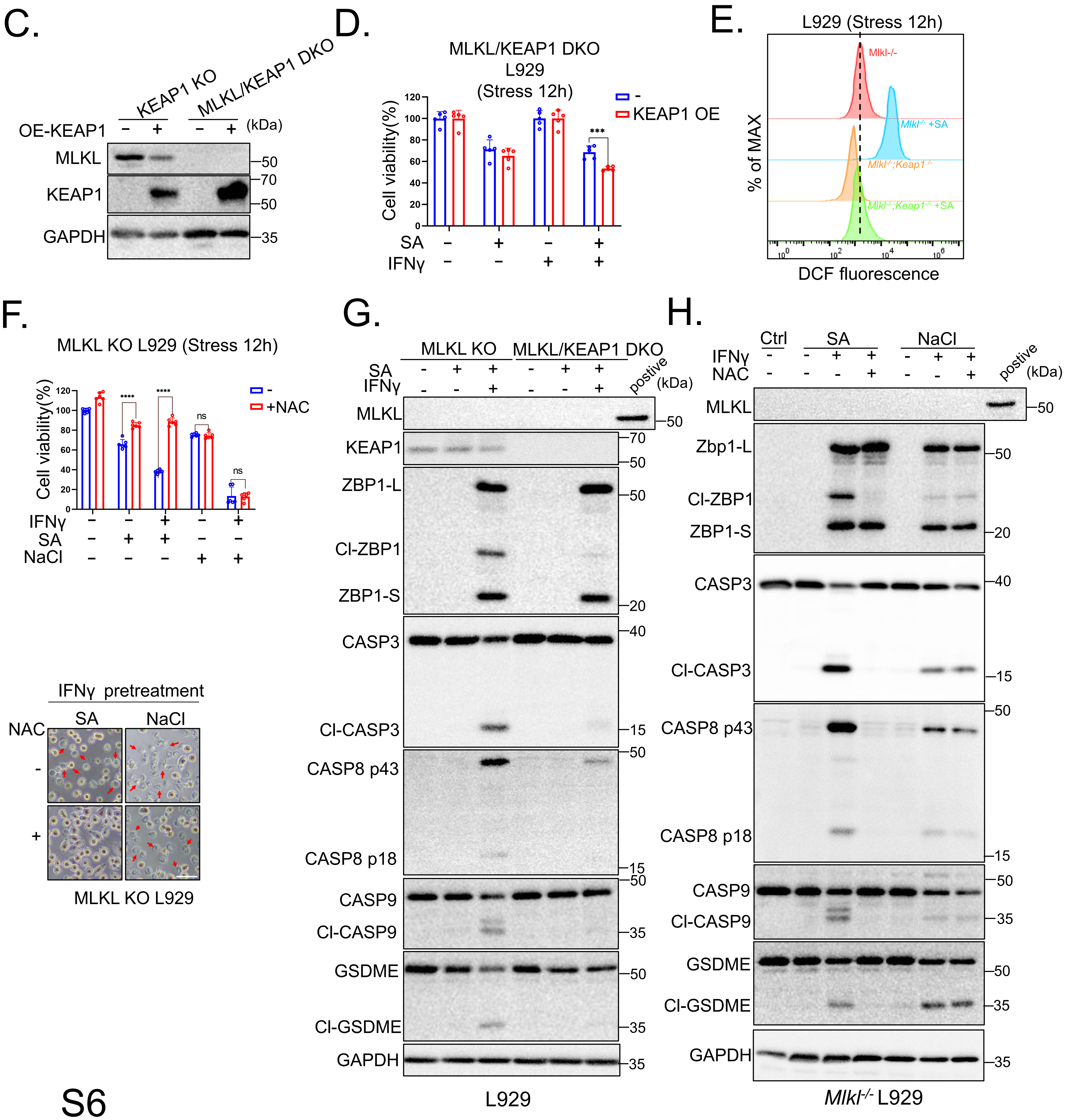

S6

Figure S6.

(A) Rank of Genes involved in stress granule formation among the recovered hits.

(B) MLKL KO or MLKL/KEAP1 DKO L929 were stressed with 100 μM sodium arsenite or 200 mM NaCl for 12 hours with or without IFNγ pretreatment. Cell viability was determined by neutral red staining (mean values ± SD; Student's t-test; ns $p > 0.1$, ****$p < 0.0001$, n = 2 independent experiments , five replicates per experiment).

(C) Immunoblotting analysis MLKL and KEAP1 in MLKL KO or MLKL/KEAP1 DKO L929 overexpressed with or without KEAP1.

(D) MLKL/KEAP1 DKO L929 cells which reconstituted with or without KEAP1 were stressed with 100 μM sodium arsenite for 12 hours with or without IFNγ pretreatment. Cell viability was determined by neutral red staining (mean values ± SD; Student's t-test; .***$p < 0.001$, n = 2 independent experiments, five replicates per experiment).

(E) DCFH-DA staining of MLKL KO or MLKL/KEAP1 DKO L929 that were stress with 100 μM sodium arsenite for 12 hours to evaluate intracellular ROS levels.

(F) Unprimed or IFNγ-primed MLKL KO L929 were stressed with 100 μM sodium arsenite or 200 mM NaCl for 12 hours with or without pretreatment of 10 mM NAC, which is a ROS scavenger, cell viability was assayed using neutral red staining. Representative pics of cells were shown, arrows indicate dead cells. (mean values ± SD; Student's t-test; ns $p > 0.1$, ****$p < 0.0001$, n = 2 independent experiments, n = 2 independent experiments, six replicates per experiment).

(G) Immunoblot analysis with indicated antibodies in MLKL KO or MLKL/KEAP1 DKO L929 stressed with 100 μM sodium arsenite for 12 hours with or without IFNγ pretreatment (n = 2 independent experiments).

(H) Immunoblot analysis of MLKL, p-MLKL, ZBP1 in unprimed or IFNγ-primed MLKL KO L929 stressed with 100 μM sodium arsenite or 200 mM NaCl for 12 hours with or without NAC pretreatment (n = 2 independent experiments).

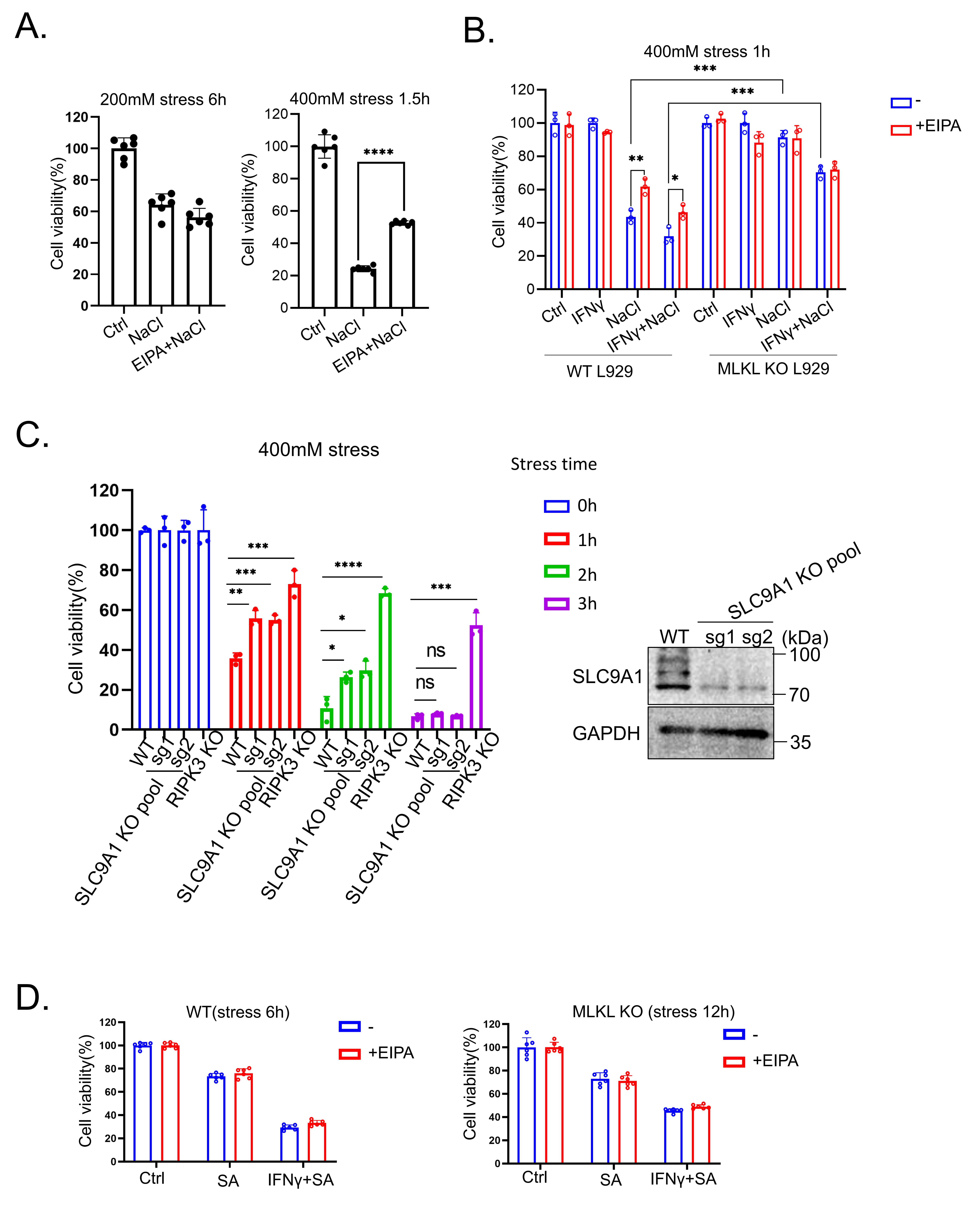
A.
200mM stress 6h
Cell viability(%)
Ctrl
NaCl
EIPA+NaCl
400mM stress 1.5h
Cell viability(%)
****
Ctrl
NaCl
EIPA+NaCl
B.
400mM stress 1h
***
***
Cell viability(%)
**
*
-
+EIPA
Ctrl
IFNγ
NaCl
IFNγ+NaCl
Ctrl
IFNγ
NaCl
IFNγ+NaCl
WT L929
MLKL KO L929
C.
400mM stress
Stress time
0h
1h
2h
3h
Cell viability(%)
***
***
**
****
*
*
***
ns
ns
WT
sg1
sg2
RIPK3 KO
SLC9A1 KO pool
SLC9A1 KO pool
WT
sg1 sg2
(kDa)
SLC9A1
GAPDH
100
70
35
D.
WT(stress 6h)
Cell viability(%)
-
+EIPA
Ctrl
SA
IFNγ+SA
MLKL KO (stress 12h)
Cell viability(%)
-
+EIPA
Ctrl
SA
IFNγ+SA

S7

Figure S7.

(A) WT L929 were treated with 200 mM or 400 mM NaCl in the presence or absence of 20 μM EIPA, cell viability was assayed using neutral red staining (mean values ± SD; Student's t-test; ****$p < 0.0001$, n = 2 independent experiments, six replicates per experiment).

(B) Unprimed or IFNγ-primed WT or MLKL KO L929 were stressed with 400 mM NaCl in the presence or absence of 20 μM EIPA. Cell viability was measured using neutral red staining (mean values ± SD; Student‘s t-test; ns $p > 0.5$ *$p < 0.05$, **$p < 0.01$, ***$p < 0.001$, ****$p < 0.0001$, n = 2 independent experiments, three replicates per experiment).

(C) Left panel: WT L929 and SLC9A1 KO pool L929 were treated with 400 mM NaCl. Cell viability was measured using neutral red staining (mean values ± SD; Student‘s t-test; *$p < 0.05$, **$p < 0.01$, ***$p < 0.001$, n = 2 independent experiments, three replicates per experiment). Right panel: Immunoblot analysis of SLC9A1 in WT or SLC9A1 KO pool L929 cells.

(D) Unprimed or IFNγ-primed WT or MLKL KO L929 were stressed with 100 μM sodium arsenite for 6 or 12 hours in the presence or absence of 20 μM EIPA. Cell viability was measured using neutral red staining (n = 2 independent experiments, six replicates per experiment).

A.

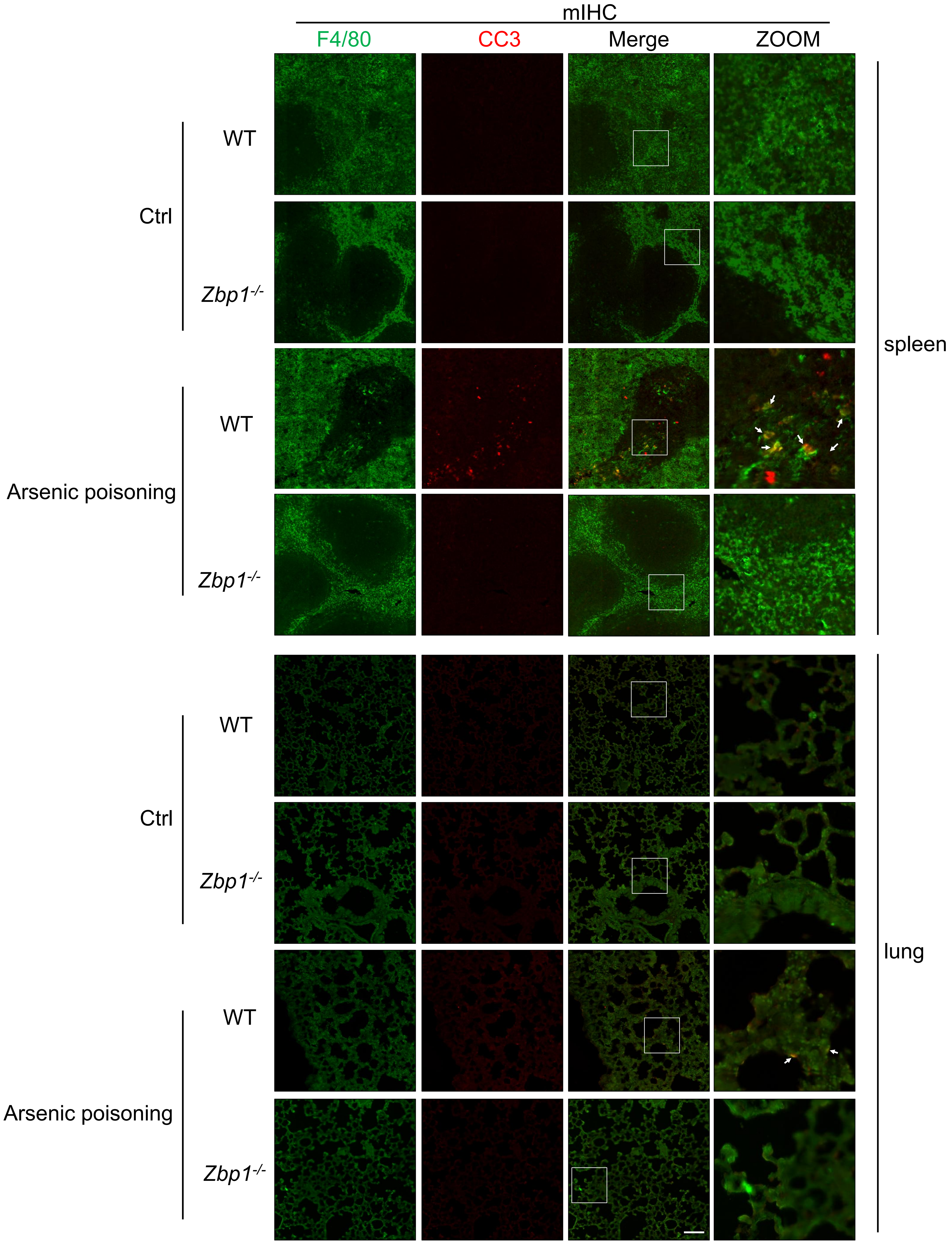

Figure S8.

(A) Multiple fluorescence immunohistochemistry F4/80 and CC3 staining of spleen and lung sections from WT or *Zbp1*$^{-/-}$ mice with or without sodium arsenite in the drinking water (n=3 mice for each group).

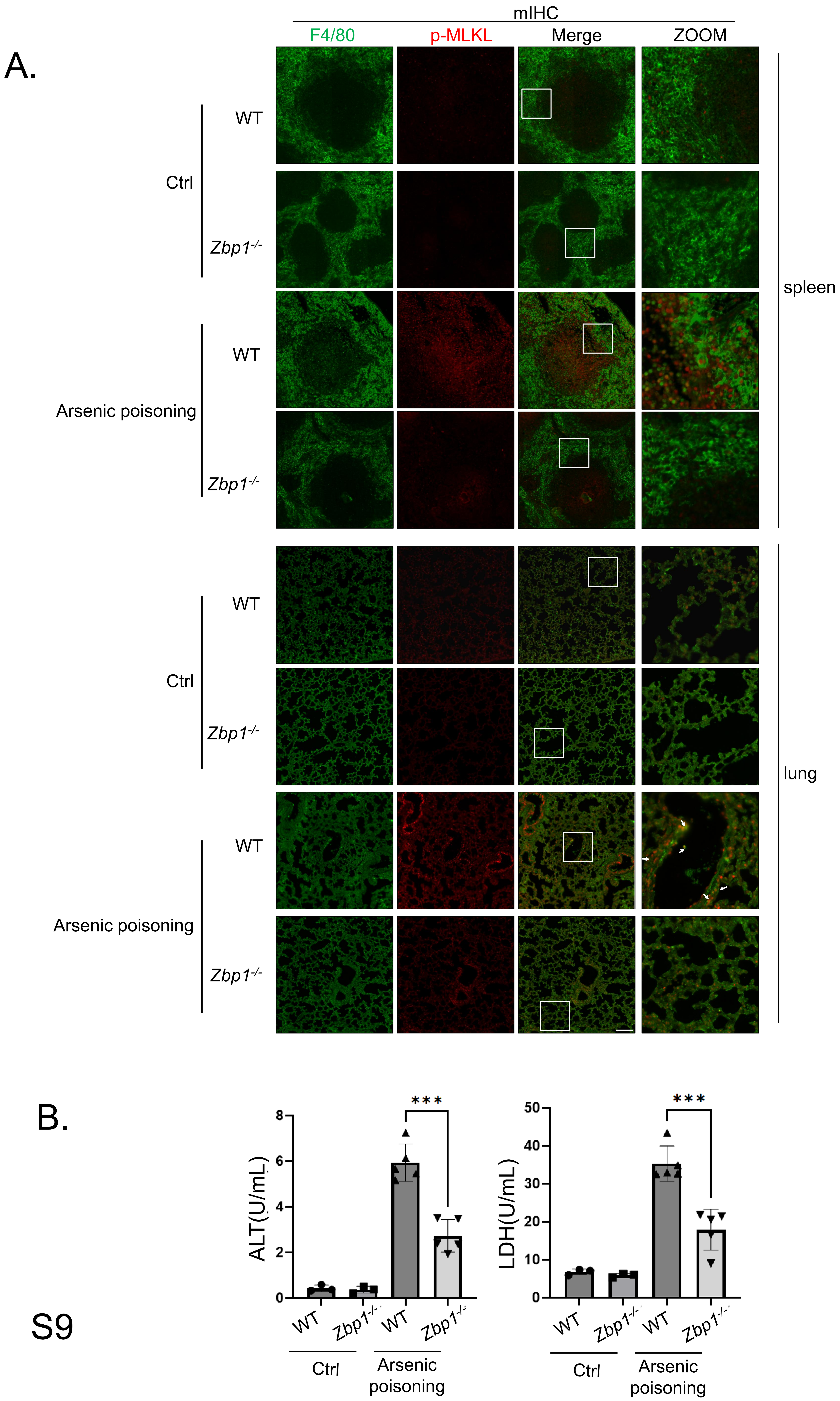

A.
mIHC
F4/80
p-MLKL
Merge
ZOOM
Ctrl
WT
Zbp1-/-
Arsenic poisoning
WT
Zbp1-/-
spleen
Ctrl
WT
Zbp1-/-
Arsenic poisoning
WT
Zbp1-/-
lung
B.
ALT(U/mL)
0
2
4
6
8
***
WT
Zbp1-/-
WT
Zbp1-/-
Ctrl
Arsenic poisoning
LDH(U/mL)
0
10
20
30
40
50
***
WT
Zbp1-/-
WT
Zbp1-/-
Ctrl
Arsenic poisoning

S9

Figure S9.

(A) Multiple fluorescence immunohistochemistry F4/80 and p-MLKL staining of spleen and lung sections from WT or *Zbp1*$^{-/-}$ mice with or without sodium arsenite in the drinking water (n=3 mice for each group).

(B) Serum levels of ALT and LDH in WT or *Zbp1*$^{-/-}$ mice were assessed with drinking water with or without sodium arsenite to determine the degree of inflammation (mean values $\pm$ SD; Student's t-test; ***$p < 0.001$, n=3 mice for each Ctrl group, n=5 mice for each Arsenic poisoning group).

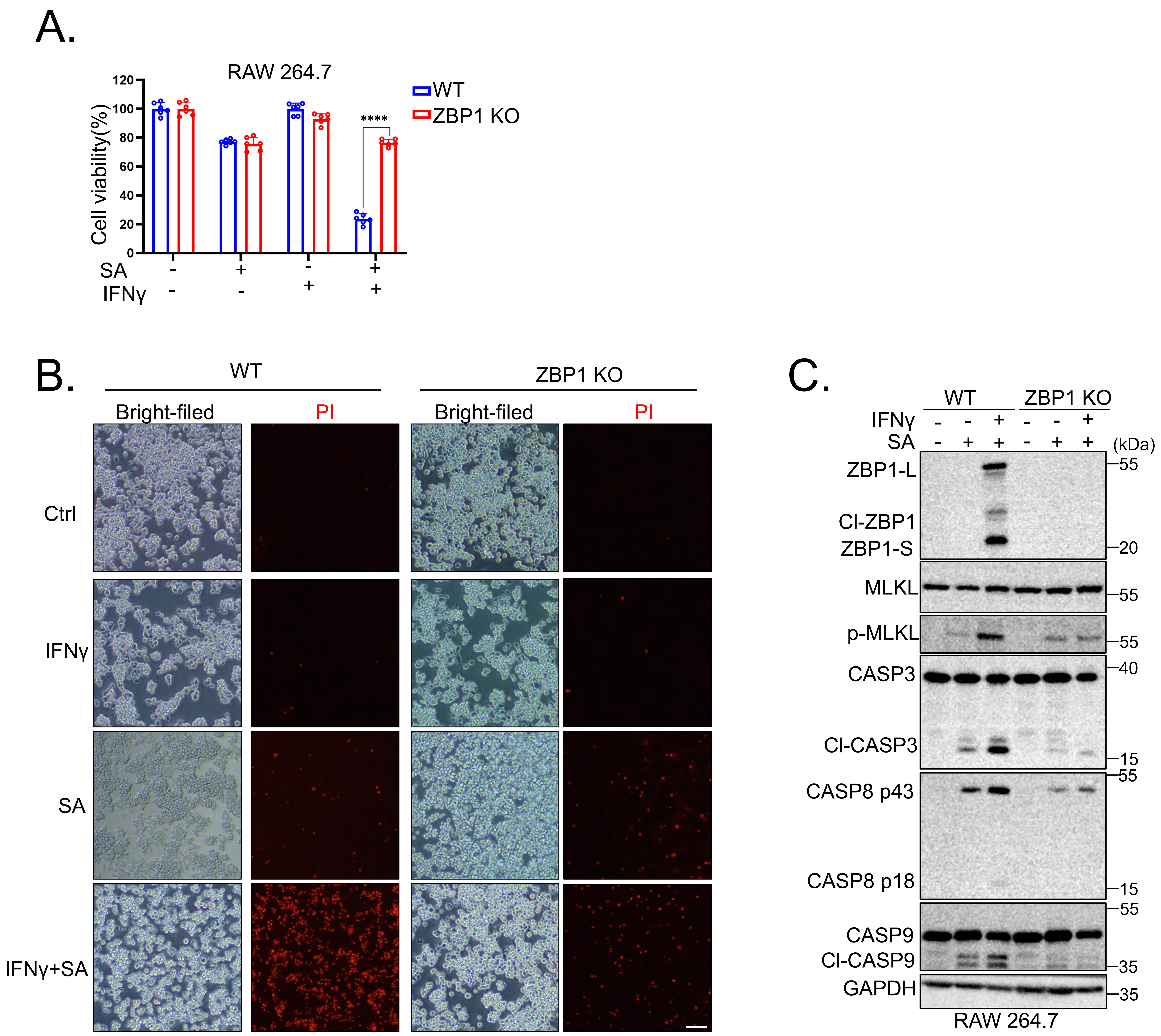

Figure S10.

(A) WT and ZBP1 KO RAW264.7 cells were treated with 100 μM sodium arsenite for 12 hours with or without IFNγ pretreatment. Cell viability was determined by neutral red staining (mean values ± SD; Student's t-test; ****$p < 0.0001$, n = 3 independent experiments, six replicates per experiment).

(B) Microscopic analysis of WT or ZBP1 KO RAW264.7 cells after PI staining for cell death induced by stress of 100 μM sodium arsenite for 12 hours with or without IFNγ pretreatment. Bar, 50 μm (n = 3 independent experiments).

(C) Immunoblot analysis with indicated antibodies in WT and ZBP1 KO RAW264.7 cells stressed with 100 μM sodium arsenite for 12 hours with or without IFNγ pretreatment (n = 2 independent experiments).
